# Development and Validation of a 6-miRNA Prognostic Signature in Spinal Chordoma

**DOI:** 10.3389/fonc.2020.556902

**Published:** 2020-10-27

**Authors:** Wei Huang, Yi-Guo Yan, Wen-Jun Wang, Zhi-Hua Ouyang, Xue-Lin Li, Tao-Lan Zhang, Xiao-Bin Wang, Bing Wang, Guo-Hua Lv, Jing Li, Ming-Xiang Zou

**Affiliations:** ^1^ Department of Spine Surgery, The First Affiliated Hospital, University of South China, Hengyang, China; ^2^ Health Management Center, The First Affiliated Hospital, University of South China, Hengyang, China; ^3^ Department of Cancer Biology, College of Medicine and Life Sciences, University of Toledo, Toledo, OH, United States; ^4^ Department of Spine Surgery, The Second Xiangya Hospital, Central South University, Changsha, China

**Keywords:** spinal chordoma, miRNA, miRNA signature, miRNA risk score, prognostic biomarkers, decision tree, survival analysis

## Abstract

**Background:**

Published data have suggested a critical role for microRNA (miRNA) expression in chordoma progression. However, most of these studies focus on single miRNA and no multi-miRNA prognostic signature has been currently established for chordoma. In this study, we sought to develop and validate a 6-miRNA risk score (miRscore) model for survival prediction.

**Methods:**

Medline, Embase, and Google scholar searches (from inception to July 20, 2018) were conducted to identify candidate miRNAs with prognostic value as per predefined criteria. Quantitative RT-PCR was used to measure miRNA levels in 114 spinal chordoma (54 in the training and 60 in the validation cohort) and 20 control specimens. Subsequently, the miRscore was built based on miRNAs data.

**Results:**

Literature searches identified six prognostic miRNAs (miR-574-3p, miR-1237-3p, miR-140-3p, miR-1, miR-155, and miR-1290) with differential expression in tumor tissues. Bioinformatical analysis revealed an important regulatory role for miR-574-3p/EGFR signaling in chordoma and showed that the target genes of these prognostic miRNAs were mainly enriched in transcription regulation, protein binding and cancer-related pathways. In both cohorts, the miRscore was associated with surrounding muscle invasion by tumor and/or other aggressive features. The miRscore model well predicted local recurrence-free survival and overall survival, which remained after adjusting for other relevant covariates. Further time-dependent receiver operating characteristics analysis in the two cohorts found that the miRscore classifier had stronger prognostic power than known clinical predictors and improved the ability of Enneking staging to predict outcomes. Importantly, recursive-partitioning analysis of both samples combined separated patients into four prognostically distinct risk subgroups for recurrence and survival (both *P* < 0.001).

**Conclusions:**

These data suggest the miRscore as a useful prognostic stratification tool in spinal chordoma and may represent an important step toward future personalized treatment of patients.

## Introduction

Chordoma is a very rare and locally aggressive malignant mesenchymal neoplasm, which has been considered to arise from remnants of the embryonic notochord (1, 2). Despite the great advance in oncology field, the current treatment of choice for chordoma is still limited. Chordoma is resistance to traditional chemotherapy or radiotherapy (3). Therefore, radical surgical excision constitutes the most important therapeutic approach for chordoma, which has been reported to provide the optimal long-term disease control when combined with postoperative adjuvant radiation (4, 5). However, even with initial maximal resection, chordoma still has high local recurrence after surgery and 40–50% of patients may even develop remote metastasis (6), which poses a large challenge for the clinical management of this tumor entity. Considering the dismal prognosis of chordoma, it is highly imperative to develop better treatment strategy aiming at improving patient survival.

Previous studies have shown that epigenetic dysfunction plays a key role in chordoma development and progression (7, 8). To be in detail, it has been demonstrated that expression of several microRNAs (miRNAs) in chordoma tissues exhibits close association with patient outcomes (8), supporting the use of miRNAs as prognostic biomarkers in chordoma. However, given that tumor initiation is driven by a complicated molecular event, single miRNA may not provide optimal information on prognosis when compared with multi-miRNA signatures (9). To overcome this, researchers have attempted to establish miRNA-based system by simultaneously integrating multiple miRNAs data in outcome prediction of cancer patients (10–13). Currently, tissue-derived miRNA models have been created for several human cancers with good predictive performance (9, 14–18), some even display obvious advantage over traditional tumor-node-metastasis classification system for survival prediction (14, 18). However, there is currently a lack of studies investigating the role of miRNA-based system for chordoma prognostic risk stratification.

Clinically, the Enneking system is commonly used to determine disease staging and guide subsequent treatment for spinal chordoma patients. However, clinical outcome varies even among patients with the same Enneking staging, implying that this system is not adequate for prognosis. Presently, there is no recognized prognostic tool for spinal chordoma in clinics. Recently, Karhade et al. (19) developed a machine learning model to provide a quantitative prediction of 5-year survival for patients with spinal chordoma using the Surveillance, Epidemiology, and End Results database. Despite large sample size, the authors only considered clinical factors in their model without simultaneously incorporating tumor molecular features, which may not offer accurate prognostic information. In the present study, we aimed to develop and validate a 6-miRNA risk score (miRscore) for spinal chordoma outcome prediction in two independent cohorts. We also attempted to establish a miRscore-based decision tree to facilitate patient-individualized therapy for neurosurgeons.

## Materials and Methods

### Patients

This study included two patient cohorts. The training cohort consisted of 54 spinal chordoma patients who underwent surgical resection at our institute between June 2002 and April 2015. This cohort has been previously reported in our studies and patients were followed-up until September 2015 (20, 21). The validation cohort recruited 23 patients from our institute (during November 2015–December 2018 period) and 37 patients from Xiangya hospital (during February 2006–October 2018 period). These patients received curative tumor excision and follow-up information was updated in April 2019. Patients selection fulfilled comparable baseline, treatment and follow-up characteristics to that of the training population (**Supplementary Table 1**) (22). Sample size in validation group was estimated in advance by a power calculation (23), and minimum of 45 patients were required to show difference with a power of 80%, β error of 0.2, and α error of 0.05. Our validation cohort had 60 patients in total, which was adequate for analysis. For all patients, clinicopathologic data were obtained from medical records and evaluated as we previously detailed (20, 21). In the training cohort, expression of PD-L1 and Ki-67, as well as the Immunoscore pattern (low: I0-I2, high: I3-I4), was directly obtained from our prior data (20, 21). In the validation cohort, immunohistochemistry was conducted to produce the above data, in which the same antibodies, dilution, staining procedure, and evaluation criteria were used as those in the training cohort to ensure comparability (20, 21).The primary outcome parameters included local relapse-free survival (LRFS) and overall survival (OS). LRFS was calculated from the date of surgery to the first local recurrence, which was considered by clinical and imaging findings and further confirmed by histologic examinations of the second surgery (24). Similarly, OS was defined as the time from tumor resection to death related to any cause. Observations were censored when a patient was tumor-free (LRFS analysis) or alive (OS analysis).

### Tissue Samples

Formalin-fixed paraffin-embedded (FFPE) tumor blocks of the 114 chordoma patients and 20 nucleus pulposus blocks (as controls) of disc herniated patients who underwent surgery during the same period were retrieved from the Department of Pathology. Control patients were randomly selected and matched for age and sex to cases from the training and validation cohort (25). Diagnosis of chordoma was made on hematoxylin and eosin-stained tissue sections by two neuropathologists per a previously published criterion (1). The study protocol was approved by our hospital ethical committee, and informed consent was obtained from each patient for publication of this study.

### Selection of Prognostic miRNAs in Spinal Chordoma

We searched MEDLINE, EMBASE and Google scholar from the inception to July 20, 2018 to identify English language studies addressing the prognostic significance of miRNAs in chordoma. The keywords combinations used were (“miR” or “miRNA” or “microRNA”) and (“chordoma” or “chordomas”). Bibliographies of the included studies and existing systematic reviews were also manually checked for any additional relevant citations missed by the above search term. Specifically, the eligible studies should provide data analyzing the relationship between miRNA expression level and clinical outcomes (such as OS, LRFS, event-free survival or progression-free survival) of patients. In addition to this restriction, only literatures in which the studied miRNA was initially selected on the basis of microarray data either from authors’ group or previously published reports were considered for further evaluation. Studies were excluded based on the following criteria: studies focused on skull base chordoma, studies not involving human, studies with fewer than 20 patients, case studies, review studies, and *in vitro* and/or *in vivo* studies with no analysis of patient outcomes.

### RNA Extraction and Quantitative RT−PCR

RNA extraction and quantitative RT−PCR were performed as we previously described (14, 26). Briefly, total RNA was isolated from 114 FFPE chordoma specimens and 20 FFPE nucleus pulposus samples using the mirVanaTM RNA isolation kit (Applied Biosystems, CA, USA) according to the manufacturer’s instructions. Total RNA (10 ng) was reversely transcribed into cDNA using the miRNA Reverse Transcription Kit (Qiagen, Dusseldorf, Germany). Quantitative PCR reactions were then conducted using a TaqMan^®^ Universal PCR Master Mix (Applied Biosystems, CA, USA). The relative expression of miRNAs was calculated by the 2^-ΔΔCT^ method. Each sample was analyzed in triplicate and U6 snRNA was used as the normalization control. As miR-155 and miR-1 have two different mature isoforms (miR-155-3p/5p and miR-1-3p/5p), the primers for these two miRNAs were determined as previously described (27, 28). The specific primers used are listed in **Supplementary Table 2**.

### Bioinformatic Analysis of Prognostic miRNAs

We used four online complementary computational databases (miRTarBase, http://mirtarbase.mbc.nctu.edu.tw/php/index.php; miRDB, http://www.mirdb.org/; miRWalk2.0, http://zmf.umm.uni-heidelberg.de/apps/zmf/mirwalk2/; TargetScan, http://www.targetscan.org/) to predict the target genes of the six prognostic miRNAs. Genes were included for analysis only when they were jointly predicted by algorithms of at least three databases. After this, we conducted gene ontology (GO) and Kyoto Encyclopedia of Genes and Genomes (KEGG) analysis to explore the main function and signaling pathways in which the annotated genes were involved. Finally, we constructed the miRNA-gene networks according to the regulatory relationships between miRNA and genes by using Cytoscape version 3.5.1 (http://www.cytoscape.org/), which is an open-source software tool developed and updated by researchers around the world to visualize biomedical networks involving proteins, genes and other interactions. We also retrieved protein-protein interaction (PPI) networks from STRING database (version 11.0, http://string-db.org).

### Construction of 6 miRNA-Based Risk Score (miRscore)

The miRscore was established as previously suggested (17, 29). First, the univariate Cox analysis was performed to screen for candidate miRNAs that were significantly correlated with OS of spinal chordoma patients. Then, candidate miRNAs were fitted into a multivariable Cox regression analysis with OS as the dependent variable to determine the contribution of each miRNA to the outcome prediction. Finally, the risk score for each patient was derived as a linear combination of the expression data for each candidate miRNAs and the corresponding multivariable Cox regression coefficients.

### Statistical Analysis

In both cohorts, quantitative data were presented as mean ± standard deviation and analyzed by Student’s t test or One-Way ANOVA test. Categorical data were analyzed by Chi-square test or Wilcoxon’s rank sum test, when appropriate. In the training cohort, the X-tile software (version 3.6.1, https://medicine.yale.edu/lab/rimm/research/software/, Rimm’s lab, Yale School of Medicine, New Haven, CT, USA) was applied to determine the cutoff points with the most significant split (the minimum *P* value approach) for continuous variables in survival analysis by regarding OS as the outcome parameter (30). To avoid overstating significance of the data, the *P* value from the log-rank test above was subsequently corrected as previously suggested (31). These cutoff values were directly used for subsequent analyses in the validation data. In the two patient cohorts, the Kaplan-Meier curve by univariate log-rank test was used to compare difference in survival probabilities between different subgroups. Similarly, multivariate Cox regression analysis was also performed on these data sets to see whether the miRscore model could independently predict patient survival, after controlling for other clinical predictors that were previously reported in literature (32–34) and/or significant in our univariate survival analysis. Time-dependent receiver operating characteristic (ROC) curve was used in both cohorts to evaluate the sensitivity and specificity of variables for survival prediction. Recursive-partitioning analysis (RPA) for creation of a decision tree was performed using the combined training and validation data to provide reference for clinical decision-making and individualized therapy (14). All statistical analyses were performed using R version 3.5.1 (R Foundation for Statistical Computing, Vienna, Austria). All tests were two-sided and a *P* value ≤ 0.05 was considered statistically significant.

## Results

### Clinical Characteristics

A total of 114 patients (54 in the training and 60 in the validation cohort) were included. Patient characteristics were recently communicated by our group (22) and summarized in **Supplementary Table 1**. Briefly, there were 35 males and 19 females in the training cohort. Of them, 43 were primary tumors and 11 were recurrent tumors. A total of 36 patients received Enneking inappropriate (EI) resection, and 18 patients underwent Enneking appropriate (EA) resection. By contrast, the validation cohort comprised 42 males and 18 females. 47 patients had primary diseases and 13 had recurrent ones. Twenty-four patients had EI resection, and 36 had EA resection. There were no significant differences for baseline characteristics between patients in training and validation cohort (**Supplementary Table 1**). All chordoma cases belonged to classic pathology type.

### Identification of Six Prognostic miRNAs Associated With Survival of Spinal Chordoma Patients

**Figure 1:**
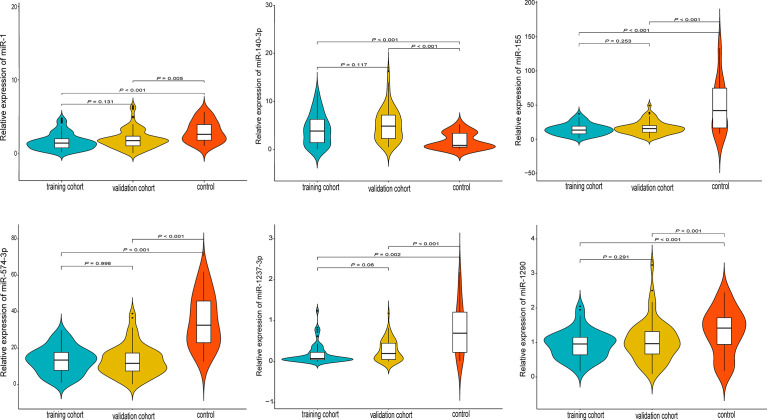
Quantitative expression levels of the six prognostic miRNAs by RT-PCR in the training, validation and control groups. All miRNAs were differentially expressed in tumor tissues compared with nucleus pulposus tissues (as controls).

A total of 122 studies were retrieved and the full-texts were fully reviewed independently by two authors (WH and MXZ) for eligibility (**Supplementary Figure 1**). After literature reviewing, six miRNAs (miR-574-3p, miR-1237-3p, miR-140-3p, miR-1, miR-155, and miR-1290) were identified to have significant association with spinal chordoma prognosis (**Supplementary Figure 1**) (20, 26–28, 35, 36). For each single miRNA, only one study reported its significant impact on spinal chordoma prognosis. Specifically, miR-140-3p (high expression in chordoma tissues compared to controls) was shown to have negative prognostic implication, while the other five miRNAs (low expression in chordoma tissues compared to controls) displayed positive association with survival of patients. These miRNAs were all initially screened by miRNA microarray and showed significantly differential expression in chordoma tissues compared with nucleus pulposus tissues (as controls) as analyzed by quantitative RT−PCR assay (**Figure 1**).

### Bioinformatic Analysis of the Six Prognostic miRNAs

**Figure 2:**
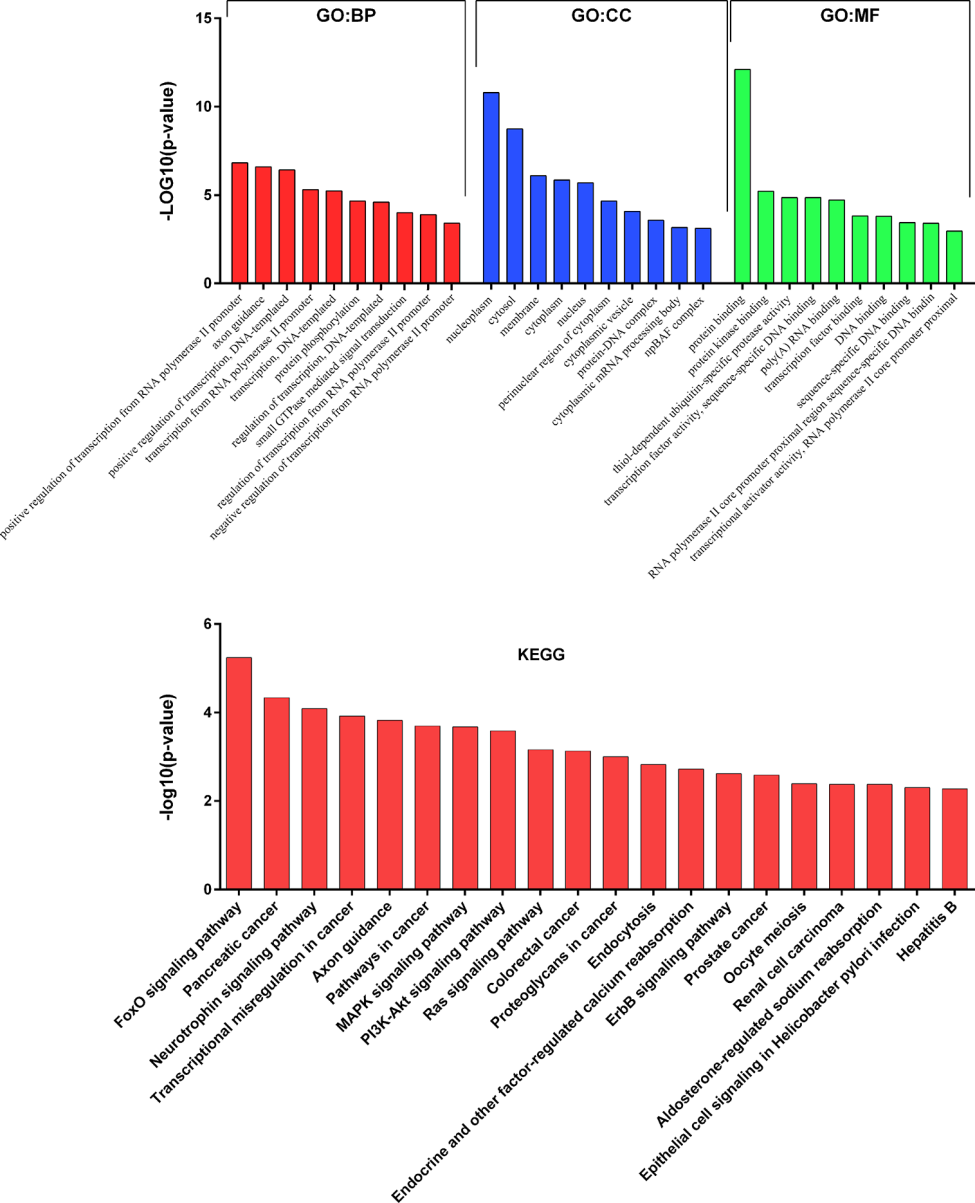
Results of GO and KEGG pathways annotation analysis of the predicted genes targeted by the six prognostic microRNAs.

**Figure 3:**
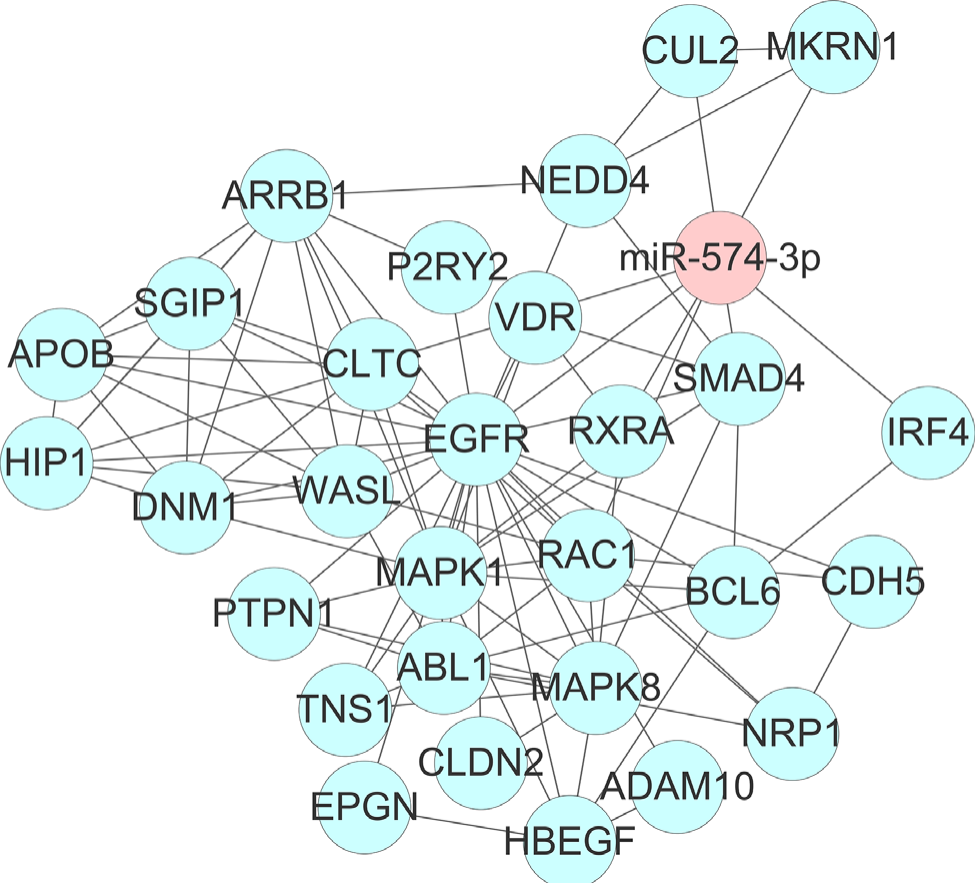
Identification of an important regulatory role for miR-574-3p-mediated signaling pathways (especially the EGFR/RAC1 pathway) in spinal chordoma by integrated analysis of both the miRNA-mRNA and protein-protein interaction networks data. miRNA, microRNA.

To get deeper insights into the biological function of the six prognostic miRNAs, we performed GO and KEGG analysis, and also generated miRNA-mRNA target regulatory and protein-protein interaction networks. It should be noted that miR-155 and miR-1 have two different mature isoforms, we therefore included all of them in computational analysis in order to obtain more comprehensive outcomes. A total of 731 nonoverlapped genes targeted by these prognostic miRNAs were identified (**Supplementary Figure 2**). The most significant GO terms corresponding to these predicted genes included regulation of gene transcription and protein binding (**Figure 2** and **Supplementary Table 3**). Pathway enrichment analysis of target genes revealed 45 pathways, including FoxO signaling, pancreatic cancer, neutrophin signaling, transcriptional misregulation in cancer, axon guidance, pathway in cancer, and other cancer-associated signaling pathways (**Figure 2** and **Supplementary Table 4**). miRNA-mRNA regulatory network showed a potentially functional link between these prognostic miRNAs (**Supplementary Figure 3**). PPI network indicated a tight connection formed by these proteins with MAPK1, MAPK8, and EGFR as the potential hub genes (**Supplementary Figure 4**). Further integrated analysis of both the miRNA-mRNA and PPI networks data identified an important regulatory role for miR-574-3p-mediated signaling pathways in chordoma, especially the EGFR pathway (**Figure 3**).

### Construction and Description of the miRscore Model

**Figure 4:**
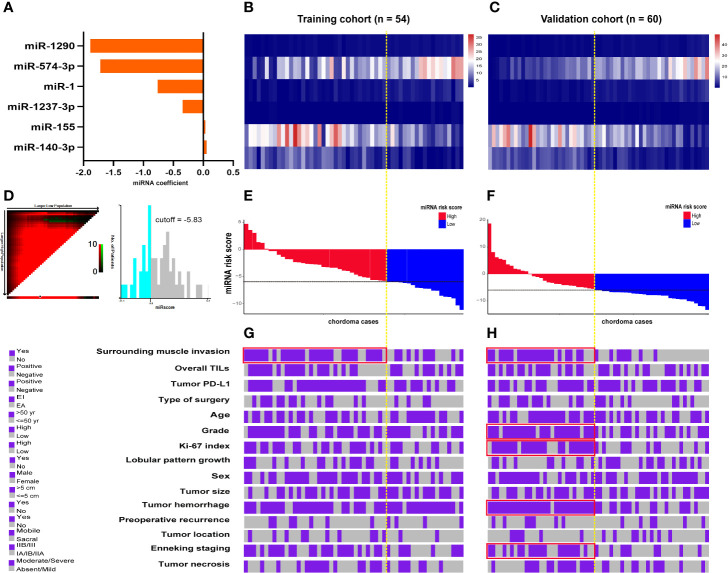
**(A)** Coefficient of each prognostic miRNA obtained by Cox regression analysis. Heatmap shows the expression data of the six miRNAs used for the miRscore construction in the training **(B)** and validation cohort **(C)**. Columns represent patients who were sorted descendingly according to their miRscore levels. **(D)** Determination of the cutoff point (-5.83) for miRscore in prognosis analysis with overall survival as the outcome parameter. Distribution of the miRscore in the training **(E)** and validation patients **(F)**. The x-axis represents patients. The intersection point of black and yellow dotted line represents the miRscore cutoff classifying patients into high and low subgroups. **(G, H)** Covariate tracks show the clinical attributes of each patient in the training and validation cohort, respectively. Red boxes indicate that the miRscore group is enriched for the indicated attribute (*P* < 0.05, Chi-square test). miRscore, microRNA risk score.

Cutoff points for the 6 prognostic miRNAs are depicted in **Supplementary Figure 5**. Patients were separated into low (≤cutoff) and high (>cutoff) groups. Univariate Kaplan-Meier analysis with the log-rank test showed that the 6 miRNAs were significantly associated with patients’ OS in both cohorts (**Supplementary Figures 6** and **7**). This was also the case for these miRNAs in the survival analysis of LRFS, except for miR-1237-3p and miR-574-3p in the training data (**Supplementary Figures 8** and **9**). Further multivariate Cox regression analysis including the 6 miRNAs data was conducted and coefficients for each miRNA were obtained (**Figure 4A**). miRscore was then calculated as a linear combination of the expression data for each miRNA (**Figures 4B, C**) and its corresponding coefficient as described above, given as follows:

$$\text{miRscore}=\Sigma [log_{2}(Expression_{miRNA}\text{) }\times \;\text{Coefficien}{\text{t}}_{miRNA}]$$

For the purpose of prognosis analysis, miRscore was also classified into low (≤-5.83) and high (>-5.83) groups according to its threshold value (**Figure 4D**). **Figures 4E, F** illustrates the distribution of miRscore data among all patients. The average value for the miRscore in the training and validation cohort was -4.16 ± 3.39 and -4.36 ± 5.62, respectively.

### Association Between miRscore and Clinicopathologic Parameters

In both cohorts, high miRscore was correlated with surrounding muscle invasion by tumors (**Figures 4G, H** and **Supplementary Tables 5** and **6**). In addition, tumors with high miRscore seemed to be more likely to have positive PD-L1 expression and advanced Enneking staging in the training cohort, although this correlation was not statistically significant (**Figures 4G, H** and **Supplementary Tables 5** and **6**). However, in the validation cohort, significant positive association was observed between the miRscore and grade, Ki-67 index, tumor hemorrhage, and the Enneking staging system (**Figures 4G, H** and **Supplementary Tables 5** and **6**).

### Association Between miRscore Classifier and Patient Outcome

**Figure 5:**
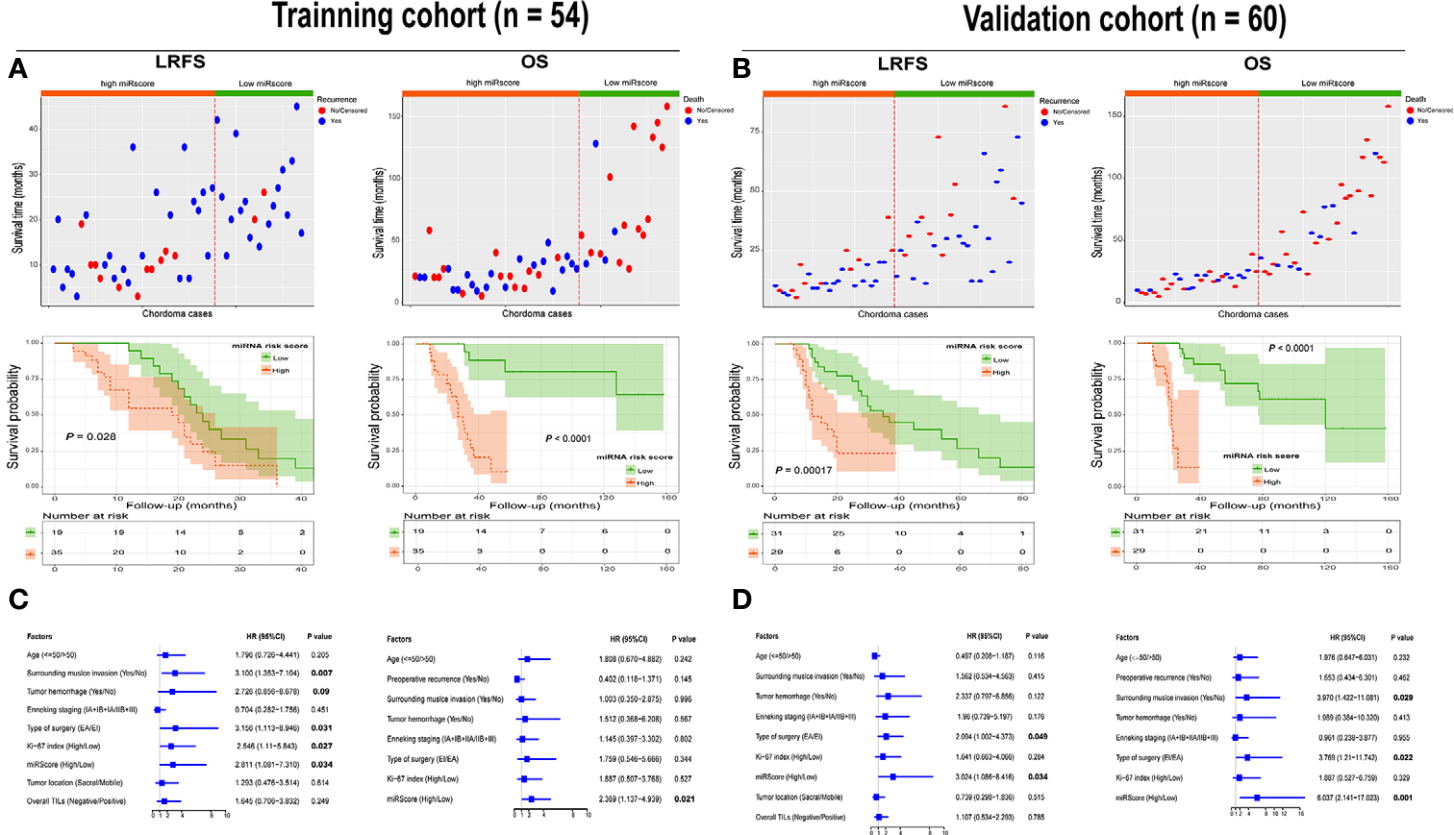
**(A)** Distribution of recurrence and survival status in chordoma patients stratified by the miRscore data in the training cohort (top). Red dotted line represents the miRscore cutoff dividing patients into high and low groups. Kaplan-Meier curve of LRFS and OS according to low or high miRscore group (middle). **(B)** Distribution of recurrence and survival status in chordoma patients stratified by the miRscore data in the training cohort (top). Red dotted line represents the miRscore cutoff dividing patients into high and low groups. Kaplan-Meier curve of LRFS and OS according to low or high miRscore group (middle). **(C)** Multivariate Cox regression model for LRFS and OS of spinal chordoma patients in the training cohort. The boxes indicate the hazard ratio, and the horizontal lines represent 95% confidence intervals. **(D)** Multivariate Cox regression model for LRFS and OS of spinal chordoma patients in the validation cohort. miRscore, microRNA risk score; LRFS, local recurrence-free survival; OS, overall survival.

In both cohorts, high miRscore appeared to correlate with a high risk of death (**Figures 5A, B**). However, low miRscore was linked to increased tumor relapse (**Figures 5A, B**). The Kaplan-Meier curve showed that patients harboring high miRscore experienced worse LRFS and OS than those with low miRscore (**Figures 5A, B**). Furthermore, multivariate Cox regression analysis adjusting for other clinical variables revealed that the miRscore was an independent predictor of both LRFS and OS (**Figures 5C, D**).

### Comparison of the miRscore Model With Six Prognostic miRNAs, the Immunoscore, Enneking Staging System, and Other Clinicopathologic Variables in Outcome Prediction

**Figure 6:**
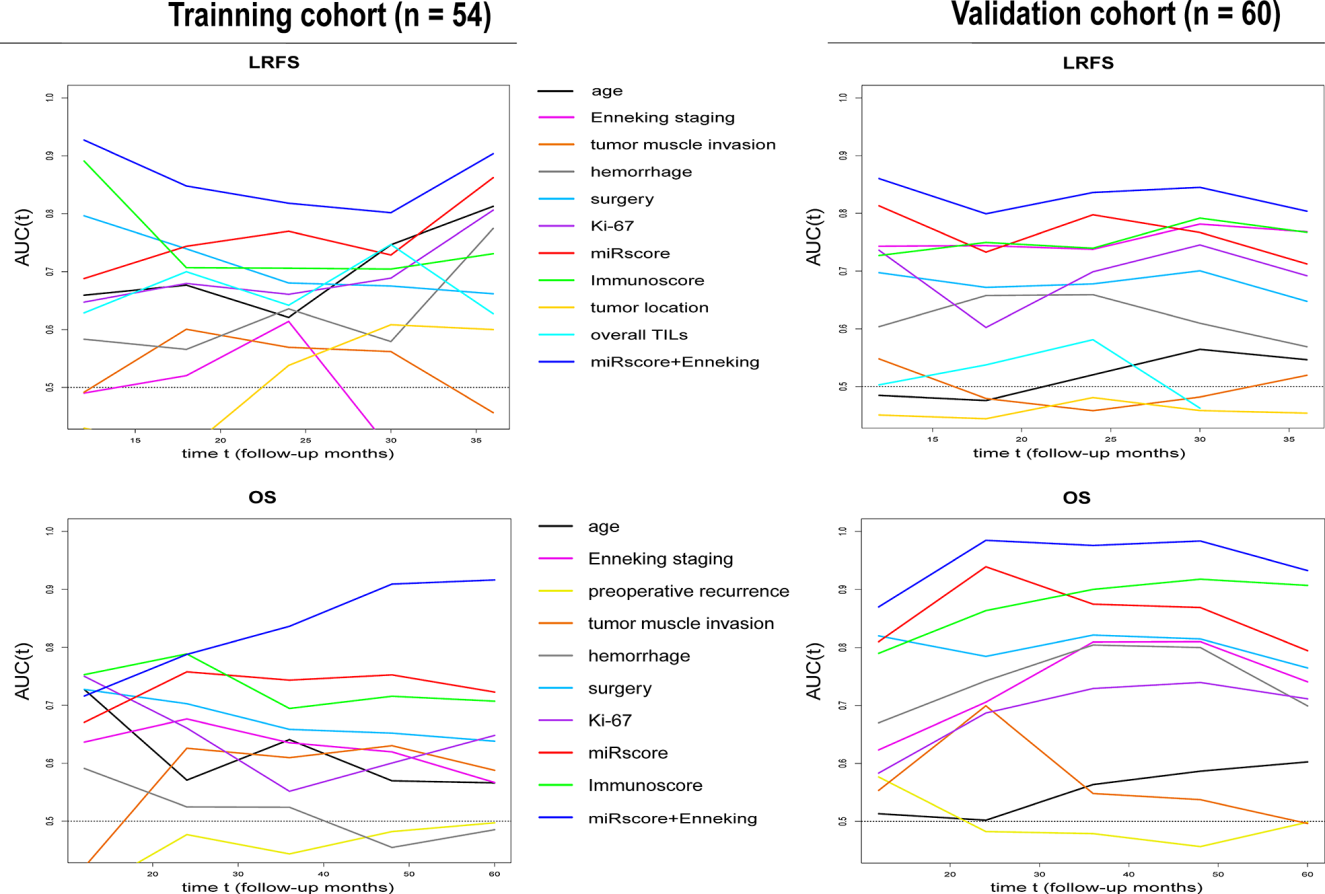
Time-dependent sensitivity and specificity derived AUCs show the predictive performance of miRscore plus Enneking staging, miRscore, Immunoscore, Enneking staging and other known clinicopathological factors for LRFS and OS prediction in the training (left) and validation samples (right) at follow-up years 1 to 3 (for LRFS) or 1-5 (for OS). AUC, area under the curve; miRscore, microRNA risk score; LRFS, local recurrence-free survival; OS, overall survival.

In both cohorts, time-dependent ROC analysis found that the miRscore model displayed comparable prognostic value to Immunoscore system in predicting survival (**Figure 6**). Moreover, the miRscore classifier provided stronger prognostic power than the Enneking staging and other classical clinical factors in LRFS and OS prediction (**Figure 6**). Importantly, combined miRscore and Enneking staging enhanced the ability of each alone for outcome prediction (**Figure 6**).

### Establishment of miRscore-Based Decision Tree for Clinical Decision-Making

**Figure 7:**
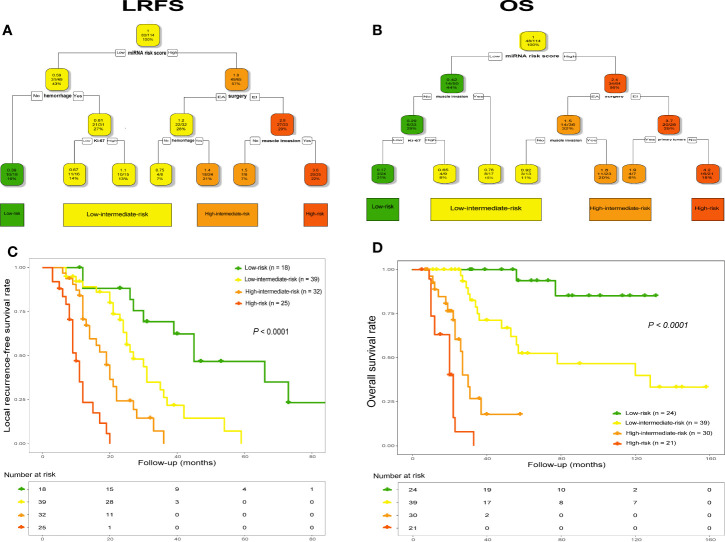
RPA tree for tumor recurrence **(A)** and survival **(B)** defined four different risk groups in the pooled data set (n = 114), including “low-risk”, “low-intermediate-risk”, “high-intermediate-risk” and “high-risk”. Each node displays the predicted probability of recurrence or death (color code low to high: green-red), the number of events for total patients, and the percentage of observations. Kaplan-Meier curves with log-rank test for LRFS **(C)** and OS **(D)** according to the four identified risk groups. LRFS, local recurrence-free survival; OS, overall survival. RPA, recursive-partitioning analysis.

Using combined data, RPA analysis identified four different risk groups for both tumor recurrence and patient death, including “low-risk”, “low-intermediate-risk”, “high-intermediate-risk” and “high-risk” groups (**Figures 7A, B**). Specifically, in analysis of LRFS, the 6-miRNA signature represented the strongest predictor together with tumor hemorrhage, type of surgery, Ki-67 index and tumor invading into surrounding muscle tissues or not (**Figure 7A**). The worst prognostic group referred to patients who received EI tumor resection and had high miRscore as well as tumor muscle invasion, while low miRscore patients without tumor hemorrhage had the best prognosis. Kaplan-Meier analysis by the log-rank test showed that the four risk groups significantly differed with regard to LRFS (**Figure 7C**). Subgroup analysis detected significant difference in LRFS time between low-risk group and low-intermediate-risk (median, 45 vs. 27 mo, *P* = 0.002), high-intermediate-risk (median, 45 vs. 19 mo, *P* < 0.001), or high-risk group (median, 45 vs. 10 mo, *P* < 0.001), and between low-intermediate-risk group and high-intermediate-risk (median, 27 vs. 19 mo, *P* = 0.001) or high-risk group (median, 27 vs. 10 mo, *P* < 0.001), as well as between high-intermediate-risk group and high-risk group (median, 19 vs. 10 mo, *P* < 0.001).

Similarly, the RPA tree showed that the miRscore signature was also the strongest parameter in terms of OS, together with type of surgery, tumor invasion of the surrounding muscle tissue, Ki-67 index and primary or recurrent tumors (**Figure 7B**). The worst prognostic group included high miRscore patients who received EI tumor resection and had recurrent disease on admission, while patients with no muscle invasion by tumor as well as low miRscore and low Ki-67 index experienced the best prognosis. Subsequent Kaplan-Meier curve revealed that the four risk groups exhibited significant difference in relation to OS (**Figure 7D**). Statistically significant differences in OS were seen between low-risk group and low-intermediate-risk (median, not estimable [NE] vs. 78 mo, *P* = 0.003), high-intermediate-risk (median, NE vs. 27 mo, *P* < 0.001), or high-risk group (median, NE vs. 20 mo, *P* < 0.001), and between low-intermediate-risk group and high-intermediate-risk (median, 78 vs. 27 mo, *P* < 0.001) or high-risk group (median, 78 vs. 20 mo, *P* < 0.001), as well as between high-intermediate-risk group and high-risk group (median, 27 vs. 20 mo, *P* = 0.002).

## Discussion

In this study, we developed a miRNA-based signature to predict the clinical outcome of spinal chordoma patients. We found that the miRscore system was closely associated with tumor surrounding muscle invasion and other aggressive clinicopathological features. The miRscore model accurately reflected survival independent of known prognostic parameters. Moreover, this signature showed good predictive performance in comparison with the Immunoscore system and other clinical factors, which could also improve predictive accuracy of the Enneking staging. Importantly, the decision tree integrating the miRscore with clinical predictors effectively separated patients into four prognostically distinct subgroups. These data suggest the use of miRscore signature as a clinical patient stratification tool in spinal chordoma and may facilitate future individualized therapy of patients.

Previous studies have demonstrated that the deregulation of miRNAs expression plays a crucial role in chordoma initiation and progression (7, 8). In support of this, our study revealed that the 6-miRNA signature was significantly correlated with adverse clinicopathological features and could independently predict survival of patients. Currently, the precise mechanism underlying the effect of miRNA signature on chordoma outcome remains undetermined. Published data have shown that the six prognostic miRNAs used for signature construction could influence chordoma prognosis by mediating expression of various target genes. For instance, Duan and colleagues found that low miR-1 expression in chordoma tissues promoted the proliferation and invasion of tumor cells by upregulation of slug (37), while miR-155 led to aggressive chordoma phenotype and poor patient survival mainly *via* regulating SOCS1 and TP53INP1 expression (28). Similarly, Wang et al. (38) proved that miR-1290 could suppress tumor cell proliferation and invasion by targeting Robo1 in chordoma. Our previous observations suggested that miR-140-3p, miR-1237-3p, and miR-574-3p could control biological behavior of chordoma cells possibly by modulating receptor tyrosine kinase pathways and expression of MMP2 and PD-L1, respectively (20, 26, 36). Collectively, these findings hint that our miRNA signature impacts chordoma outcome through complicated molecular events. This speculation can be corroborated by our subsequent bioinformatic analysis, showing that target genes of the six miRNAs were involved in various GO terms and signaling pathways related to cancer.

In addition, our computational analysis also disclosed a key regulatory role for miR-574-3p/EGFR pathway in chordoma. We previously reported that miR-574-3p could influence immune profiles of chordoma possibly by targeting PD-L1 (20). Moreover, recent data have provided evidence for EGFR/Ras/Mek signaling to control PD-L1 expression in cancers (39, 40). Taken together, these outcomes further highlight the clinical significance of immune microenvironment in chordoma (21, 41, 42) and suggest a complete miR-574-3p/EGFR/Ras/Mek/PD-L1 signaling network involved in chordoma development. This finding deserves further investigation and may have implication for sole or combination immunotherapy approaches for chordoma treatment.

Our study found that the miRscore system could effectively portend chordoma outcome independent of known parameters. Moreover, this miRNA-based signature had better prognostic power than the traditional Enneking system and also possessed comparable predictive ability to that of the Immunoscore system. Importantly, combined miRscore and Enneking staging model significantly improved predictive accuracy of each alone in both cohorts. Altogether, these results indicate that adding miRNA expression data into chordoma prognostic grading system is necessary in order to produce a reliable disease model and then guide therapeutic optimization. Preceding reports have suggested that the Enneking system as an indicator of anatomic features of tumors is not adequate to inform chordoma prognosis (21). Further, it has been determined that the immune microenvironment reflective of tumor bioimmunological properties plays a pivotal role in chordoma progression (20, 21, 41). Besides these data, recent studies have implied that radiological findings on magnetic resonance imaging are also related to chordoma outcome (43, 44). As the miRscore signature represents different molecular features from those above, future prognostic model considering all these aspects may prove a more accurate and reliable tool for risk stratification in spinal chordoma.

Interesting, we also found that the miRscore-based decision tree could effectively split patients into four different risk subgroups, with significantly distinct survival. Of note, in this setting, the miRscore was the most important element among other clinical parameters for both disease recurrence and death. These data reiterate the clinical relevance of the 6-miRNA signature in spina chordoma and offer an approach for more detailed, clinically meaningful stratification of patients, which can be useful in identifying cases with advanced disease and then making treatment decisions timely.

## Limitations

Additional studies are needed to optimize the miRscore calculation for clinical utility. Besides, it should be noted that parts of our validation datasets were from the same institute as the training cohort, which might compromise the generalization of our findings. We did this because this intervention could enable us to provide more reliable results by analyzing the current validation data as enough sample size was reached to obtain statistically significant difference. But before translating the miRscore model into clinical practice, prospective external validation studies with large sample sizes especially from a different institute are still needed to corroborate our current data in the future. In addition, published data have suggested that many other miRNAs may impact patient outcomes in spinal chordoma (7). Our future study will further evaluate the eligibility for adding other miRNAs (such as miR-219-5p and miR-31) into the miRscore model and assessed their effect on spinal chordoma prognosis (45, 46). Finally, our study is a correlation study in nature. More studies will be required to clarify how the miRscore signature affects clinical outcome of patients.

## Conclusions

The presented study identified a six-miRNA molecular signature that was associated with aggressive clinicopathological features and survival of spinal chordoma patients. The miRscore model had good predictive performance and also compensated the deficiency of Enneking system for survival prediction. The integration of the miRscore with known prognostically clinical parameters in decision tree allowed the definition of four subgroups with significantly different survival. These data suggest the miRscore as a useful prognostic stratification tool in spinal chordoma and may represent an important step toward future personalized treatment of patients.

## Data Availability Statement

All datasets presented in this study are included in the article/**Supplementary Material**.

## Ethics Statement

The study protocol was approved by the Institutional Review Board at the First Affiliated Hospital, University of South China, Hunan, China. Written informed consent was obtained from each patient for publication of this study.

## Author Contributions

All authors participated in data acquisition. WH, G-HL, JL, and M-XZ contributed to the conception and design of the study. WH, Y-GY, W-JW, Z-HO, BW, and M-XZ did the data analysis and interpretation. WH, X-LL, T-LZ, and X-BW performed the experiments. WH, Z-HO, T-LZ, X-BW, and M-XZ contributed to drafting and revision of the manuscript. All authors contributed to the article and approved the submitted version.

## Funding

This work was supported by the National Natural Science Foundation of China (81802211 to X-BW and 81871821 to JL), Natural Science Foundation of Hunan Province (2019JJ50542 to T-LZ and 2018JJ3738 to X-BW), and Project for Clinical Research of Hunan Provincial Health Commission (20201978 to T-LZ and 20201956 to M-XZ).

## Conflict of Interest

The authors declare that the research was conducted in the absence of any commercial or financial relationships that could be construed as a potential conflict of interest.

## Abbreviations

miRNA, microRNA; miRscore, miRNA risk score; FFPE, formalin-fixed paraffin-embedded; LRFS, local relapse-free survival; OS, overall survival; GO, gene ontology; KEGG, Kyoto Encyclopedia of Genes and Genomes; ROC, receiver operating characteristic; RPA, Recursive-partitioning analysis; EA, Enneking appropriate; EI, Enneking inappropriate; PD-L1, programmed cell death-1 ligand 1; EGFR, epidermal growth factor receptor; PPI, protein-protein interaction; NE, not estimable.
