# Supplementary material for: Development and Validation of a 6-miRNA Prognostic Signature in Spinal Chordoma
Source: Front Oncol. 2020 Oct 27;10:556902. doi: 10.3389/fonc.2020.556902 (PMC7656123; doi:10.3389/fonc.2020.556902)
Supplement: Supplementary file 1 [file DataSheet_1.doc]

**Supplementary Table 1** Comparison of patient characteristics between the training and validation cohort

| Characteristic | Training cohort (n = 54) | Validation cohort (n = 60) | Test statistic | *P-*value |
| --- | --- | --- | --- | --- |
| Age (years) |  |  |  |  |
| ≤ 50 | 23 (42.6%) | 20 (33.3%) | 1.037 | 0.308 |
| > 50 | 31 (57.4%) | 40 (66.7%) |  |  |
| Sex |  |  |  |  |
| Male | 35 (64.8%) | 42 (70%) | 0.349 | 0.555 |
| Female | 19 (35.2%) | 18 (30%) |  |  |
| Tumor size |  |  |  |  |
| ≤ 5 cm | 21 (38.9%) | 22 (36.7%) | 0.060 | 0.807 |
| > 5 cm | 33 (61.1%) | 38 (63.3%) |  |  |
| Tumor location |  |  |  |  |
| Sacral vertebra | 42 (77.8%) | 45 (75%) | 0.121 | 0.728 |
| Mobile spine | 12 (22.2%) | 15 (25%) |  |  |
| Surrounding  muscle invasion |  |  |  |  |
| Yes | 36 (66.7%) | 31 (51.7%) | 2.639 | 0.104 |
| No | 18 (33.3%) | 29 (48.3%) |  |  |
| Preoperative recurrence |  |  |  |  |
| Yes | 11 (20.4%) | 13 (21.7%) | 0.029 | 0.865 |
| No | 43 (79.6%) | 47 (78.3%) |  |  |
| Grade |  |  |  |  |
| High | 38 (70.4%) | 42 (70%) | 0.002 | 0.966 |
| Low | 16 (29.6%) | 18 (30%) |  |  |
| Enneking staging |  |  |  |  |
| IA + IB + IIA | 23 (42.6%) | 33 (55%) | 1.751 | 0.186 |
| IIB + III | 31 (57.4%) | 27 (45%) |  |  |
| Type of resection |  |  |  |  |
| EI | 18 (33.3%) | 24 (40%) | 0.543 | 0.461 |
| EA | 36 (66.7%) | 36 (60%) |  |  |
| Tumor hemorrhage |  |  |  |  |
| No | 10 (18.5%) | 17 (28.3%) | 1.515 | 0.218 |
| Yes | 44 (81.5%) | 43 (71.7%) |  |  |
| Tumor necrosis |  |  |  |  |
| Absent + Mild | 31 (57.4%) | 33 (55%) | 0.067 | 0.796 |
| Moderate + Severe | 23 (42.6%) | 27 (45%) |  |  |
| Ki-67 index |  |  |  |  |
| Low | 25 (46.3%) | 26 (43.3%) | 0.101 | 0.751 |
| High | 29 (53.7%) | 34 (56.7%) |  |  |
| Tumor PD-L1 |  |  |  |  |
| Positive | 37 (68.5%) | 40 (66.7%) | 0.044 | 0.833 |
| Negative | 17 (31.5%) | 20 (33.3%) |  |  |
| Lobular pattern growth |  |  |  |  |
| Yes | 21 (38.9%) | 25 (41.7%) | 0.091 | 0.763 |
| No | 33 (61.1%) | 35 (58.3%) |  |  |
| Overall TILs |  |  |  |  |
| Positive | 31 (57.4%) | 34 (56.7%) | 0.006 | 0.936 |
| Negative | 23 (42.6%) | 26 (43.3%) |  |  |
| Follow-up duration (months) | 42.4 ± 38.9 | 43.4 ± 36.9 | -0.145 | 0.885 |

EI, Enneking inappropriate; EA, Enneking appropriate; TILs, tumor-infiltrating lymphocytes; PD-L1,

programmed cell death-1 ligand 1.

**Supplementary Table 2** List of the specific primers for six prognostic microRNAs

| Gene | Forward | Reverse |
| --- | --- | --- |
| miR-574-3p | 5’-GGCGCACGCTCATGCACACA-3’ | 5’-GTGCAGGGTCCGAGGT-3’ |
| miR-1237-3p | 5’-TCCTTCTGCTCCGTCCCCCAG-3’ | 5’-GTGCAGGGTCCGAGGT-3’ |
| miR-140-3p | 5’-TACCACAGGGTAGAACCACGG-3’ | 5’-GTGCAGGGTCCGAGGT-3’ |
| miR-1290 | 5’-CAGTGCTGGATTTTTGGAT-3’ | 5’-GTGCAGGGTCCGAGGT-3’ |
| miR-1 | 5’-TGGAATGTAAAGAAGTATGTAT-3’ | 5’-GTGCAGGGTCCGAGGT-3’ |
| miR-155 | 5’-CTCCTACATATTAGCATTAAC-3’ | 5’-GTGCAGGGTCCGAGGT-3’ |
| U6 | 5’-CTCGCTTCGGCAGCACA-3’ | 5’-AACGCTTCACGAATTTGCGT-3’ |

**Supplementary Table 3** Results of GO analysis of the predicted target genes affected by the six prognostic miRNAs

| **Term (biological process)** | **Count** | **%** | **P-Value** | **FDR** |
| --- | --- | --- | --- | --- |
| positive regulation of transcription from RNA polymerase II promoter | 7.30E+01 | 10 | 1.50E-07 | 4.50E-04 |
| axon guidance | 2.30E+01 | 3.2 | 2.50E-07 | 3.60E-04 |
| positive regulation of transcription, DNA-templated | 4.60E+01 | 6.3 | 3.80E-07 | 3.80E-04 |
| transcription from RNA polymerase II promoter | 4.30E+01 | 5.9 | 4.90E-06 | 3.60E-03 |
| transcription, DNA-templated | 1.15E+02 | 15.8 | 5.90E-06 | 3.50E-03 |
| protein phosphorylation | 3.80E+01 | 5.2 | 2.20E-05 | 1.10E-02 |
| regulation of transcription, DNA-templated | 9.10E+01 | 12.5 | 2.50E-05 | 1.10E-02 |
| small GTPase mediated signal transduction | 2.40E+01 | 3.3 | 1.00E-04 | 3.60E-02 |
| regulation of transcription from RNA polymerase II promoter | 3.50E+01 | 4.8 | 1.30E-04 | 4.10E-02 |
| negative regulation of transcription from RNA polymerase II promoter | 4.80E+01 | 6.6 | 3.90E-04 | 1.10E-01 |
| regulation of cell motility | 7.00E+00 | 1 | 6.20E-04 | 1.50E-01 |
| positive regulation of mitotic cell cycle | 7.00E+00 | 1 | 6.20E-04 | 1.50E-01 |
| positive regulation of gene expression | 2.30E+01 | 3.2 | 6.30E-04 | 1.40E-01 |
| ventricular cardiac muscle cell differentiation | 4.00E+00 | 0.5 | 1.10E-03 | 2.20E-01 |
| regulation of endocytosis | 7.00E+00 | 1 | 1.10E-03 | 2.10E-01 |
| positive regulation of GTPase activity | 3.80E+01 | 5.2 | 1.50E-03 | 2.60E-01 |
| actin cytoskeleton organization | 1.40E+01 | 1.9 | 1.70E-03 | 2.70E-01 |
| positive regulation of cell migration | 1.70E+01 | 2.3 | 2.30E-03 | 3.30E-01 |
| response to stress | 9.00E+00 | 1.2 | 2.40E-03 | 3.30E-01 |
| histone H4-K8 acetylation | 5.00E+00 | 0.7 | 2.90E-03 | 3.60E-01 |
| histone H4-K5 acetylation | 5.00E+00 | 0.7 | 2.90E-03 | 3.60E-01 |
| response to wounding | 9.00E+00 | 1.2 | 3.00E-03 | 3.60E-01 |
| endocytosis | 1.40E+01 | 1.9 | 3.10E-03 | 3.50E-01 |
| regulation of protein localization | 8.00E+00 | 1.1 | 3.90E-03 | 4.10E-01 |
| negative regulation of transcription, DNA-templated | 3.30E+01 | 4.5 | 4.10E-03 | 4.10E-01 |
| cellular response to hypoxia | 1.10E+01 | 1.5 | 4.20E-03 | 4.10E-01 |
| covalent chromatin modification | 1.20E+01 | 1.6 | 4.60E-03 | 4.20E-01 |
| positive regulation of cell proliferation | 3.10E+01 | 4.3 | 5.10E-03 | 4.40E-01 |
| cellular sodium ion homeostasis | 5.00E+00 | 0.7 | 5.60E-03 | 4.60E-01 |
| positive regulation of pri-miRNA transcription from RNA polymerase II promoter | 5.00E+00 | 0.7 | 6.70E-03 | 5.10E-01 |
| histone H4-K16 acetylation | 5.00E+00 | 0.7 | 6.70E-03 | 5.10E-01 |
| peptidyl-tyrosine phosphorylation | 1.40E+01 | 1.9 | 7.00E-03 | 5.10E-01 |
| protein stabilization | 1.30E+01 | 1.8 | 7.00E-03 | 5.00E-01 |
| negative regulation of ERK1 and ERK2 cascade | 8.00E+00 | 1.1 | 7.10E-03 | 4.90E-01 |
| post-embryonic development | 9.00E+00 | 1.2 | 7.40E-03 | 5.00E-01 |
| hemopoiesis | 8.00E+00 | 1.1 | 7.80E-03 | 5.00E-01 |
| phosphatidylinositol-mediated signaling | 1.10E+01 | 1.5 | 8.40E-03 | 5.20E-01 |
| Golgi reassembly | 3.00E+00 | 0.4 | 8.60E-03 | 5.20E-01 |
| secretory columnal luminar epithelial cell differentiation involved in prostate glandular acinus development | 3.00E+00 | 0.4 | 8.60E-03 | 5.20E-01 |
| regulation of transforming growth factor beta2 production | 3.00E+00 | 0.4 | 8.60E-03 | 5.20E-01 |
| cell proliferation | 2.50E+01 | 3.4 | 9.30E-03 | 5.40E-01 |
| dopaminergic neuron differentiation | 5.00E+00 | 0.7 | 9.60E-03 | 5.40E-01 |
| cell-cell adhesion | 2.00E+01 | 2.7 | 1.00E-02 | 5.50E-01 |
| cell morphogenesis | 8.00E+00 | 1.1 | 1.00E-02 | 5.40E-01 |
| positive regulation of protein phosphorylation | 1.20E+01 | 1.6 | 1.10E-02 | 5.60E-01 |
| NLS-bearing protein import into nucleus | 5.00E+00 | 0.7 | 1.10E-02 | 5.60E-01 |
| protein transport | 2.60E+01 | 3.6 | 1.20E-02 | 5.80E-01 |
| multicellular organism growth | 9.00E+00 | 1.2 | 1.30E-02 | 5.80E-01 |
| regulation of autophagy | 7.00E+00 | 1 | 1.30E-02 | 5.80E-01 |
| protein K48-linked deubiquitination | 5.00E+00 | 0.7 | 1.30E-02 | 5.80E-01 |
| collateral sprouting | 3.00E+00 | 0.4 | 1.40E-02 | 6.00E-01 |
| MAPK cascade | 1.90E+01 | 2.6 | 1.50E-02 | 6.00E-01 |
| protein deubiquitination | 1.00E+01 | 1.4 | 1.50E-02 | 6.10E-01 |
| substrate adhesion-dependent cell spreading | 6.00E+00 | 0.8 | 1.50E-02 | 6.10E-01 |
| cholesterol biosynthetic process | 6.00E+00 | 0.8 | 1.50E-02 | 6.10E-01 |
| positive regulation of B cell proliferation | 6.00E+00 | 0.8 | 1.70E-02 | 6.40E-01 |
| positive regulation of viral genome replication | 5.00E+00 | 0.7 | 1.70E-02 | 6.40E-01 |
| regulation of nitric-oxide synthase activity | 5.00E+00 | 0.7 | 1.70E-02 | 6.40E-01 |
| protein autophosphorylation | 1.40E+01 | 1.9 | 1.70E-02 | 6.30E-01 |
| nervous system development | 2.00E+01 | 2.7 | 1.80E-02 | 6.30E-01 |
| rhythmic process | 7.00E+00 | 1 | 1.80E-02 | 6.30E-01 |
| chromatin remodeling | 9.00E+00 | 1.2 | 1.90E-02 | 6.40E-01 |
| regulation of cell communication by electrical coupling | 3.00E+00 | 0.4 | 2.10E-02 | 6.70E-01 |
| epidermal growth factor receptor signaling pathway | 7.00E+00 | 1 | 2.10E-02 | 6.70E-01 |
| cell cycle arrest | 1.20E+01 | 1.6 | 2.20E-02 | 6.80E-01 |
| signal transduction | 6.00E+01 | 8.2 | 2.30E-02 | 6.80E-01 |
| definitive hemopoiesis | 4.00E+00 | 0.5 | 2.30E-02 | 6.80E-01 |
| muscle organ development | 9.00E+00 | 1.2 | 2.30E-02 | 6.70E-01 |
| receptor internalization | 6.00E+00 | 0.8 | 2.50E-02 | 7.00E-01 |
| histone H3 acetylation | 6.00E+00 | 0.8 | 2.50E-02 | 7.00E-01 |
| ubiquitin-dependent protein catabolic process | 1.40E+01 | 1.9 | 2.60E-02 | 7.10E-01 |
| nucleosome disassembly | 4.00E+00 | 0.5 | 2.70E-02 | 7.10E-01 |
| dentate gyrus development | 4.00E+00 | 0.5 | 2.70E-02 | 7.10E-01 |
| regulation of anion transmembrane transport | 4.00E+00 | 0.5 | 2.70E-02 | 7.10E-01 |
| intracellular signal transduction | 2.50E+01 | 3.4 | 2.70E-02 | 7.10E-01 |
| positive regulation of MAP kinase activity | 7.00E+00 | 1 | 2.70E-02 | 7.10E-01 |
| regulation of macroautophagy | 6.00E+00 | 0.8 | 2.80E-02 | 7.10E-01 |
| synaptic transmission, GABAergic | 3.00E+00 | 0.4 | 2.80E-02 | 7.10E-01 |
| cellular response to phorbol 13-acetate 12-myristate | 3.00E+00 | 0.4 | 2.80E-02 | 7.10E-01 |
| angiogenesis | 1.60E+01 | 2.2 | 2.90E-02 | 7.10E-01 |
| endoplasmic reticulum unfolded protein response | 6.00E+00 | 0.8 | 3.00E-02 | 7.20E-01 |
| activation of protein kinase activity | 6.00E+00 | 0.8 | 3.00E-02 | 7.20E-01 |
| cellular response to insulin stimulus | 8.00E+00 | 1.1 | 3.00E-02 | 7.20E-01 |
| regulation of actin cytoskeleton reorganization | 4.00E+00 | 0.5 | 3.10E-02 | 7.30E-01 |
| cell fate commitment | 6.00E+00 | 0.8 | 3.30E-02 | 7.40E-01 |
| regulation of actin cytoskeleton organization | 6.00E+00 | 0.8 | 3.50E-02 | 7.60E-01 |
| protein localization to plasma membrane | 7.00E+00 | 1 | 3.60E-02 | 7.60E-01 |
| retinal ganglion cell axon guidance | 4.00E+00 | 0.5 | 3.60E-02 | 7.60E-01 |
| ion transmembrane transport | 1.50E+01 | 2.1 | 3.60E-02 | 7.60E-01 |
| germinal center formation | 3.00E+00 | 0.4 | 3.60E-02 | 7.50E-01 |
| positive regulation by host of viral genome replication | 3.00E+00 | 0.4 | 3.60E-02 | 7.50E-01 |
| response to hypoxia | 1.30E+01 | 1.8 | 3.70E-02 | 7.60E-01 |
| negative regulation of cell proliferation | 2.40E+01 | 3.3 | 3.80E-02 | 7.60E-01 |
| positive regulation of peptidyl-tyrosine phosphorylation | 8.00E+00 | 1.1 | 4.10E-02 | 7.80E-01 |
| clathrin-mediated endocytosis | 4.00E+00 | 0.5 | 4.10E-02 | 7.80E-01 |
| cellular response to transforming growth factor beta stimulus | 6.00E+00 | 0.8 | 4.10E-02 | 7.80E-01 |
| single organismal cell-cell adhesion | 9.00E+00 | 1.2 | 4.40E-02 | 7.90E-01 |
| positive regulation of cell growth | 8.00E+00 | 1.1 | 4.60E-02 | 8.00E-01 |
| positive regulation of transcription from RNA polymerase II promoter involved in cellular response to chemical stimulus | 3.00E+00 | 0.4 | 4.60E-02 | 8.00E-01 |
| regulation of vesicle-mediated transport | 3.00E+00 | 0.4 | 4.60E-02 | 8.00E-01 |
| regulation of bone resorption | 3.00E+00 | 0.4 | 4.60E-02 | 8.00E-01 |
| endothelial cell chemotaxis | 3.00E+00 | 0.4 | 4.60E-02 | 8.00E-01 |
| Fc-epsilon receptor signaling pathway | 1.30E+01 | 1.8 | 4.60E-02 | 8.00E-01 |
| response to ATP | 4.00E+00 | 0.5 | 4.70E-02 | 8.00E-01 |
| positive regulation of apoptotic process | 1.90E+01 | 2.6 | 4.70E-02 | 8.00E-01 |
| **RT (cellular component)** | **Count** | **%** | ***P*-Value** | **FDR** |
| nucleoplasm | 172 | 23.6 | 1.60E-11 | 8.10E-09 |
| cytosol | 188 | 25.8 | 1.80E-09 | 4.60E-07 |
| membrane | 127 | 17.4 | 8.10E-07 | 1.40E-04 |
| cytoplasm | 254 | 34.8 | 1.40E-06 | 1.90E-04 |
| nucleus | 261 | 35.8 | 2.00E-06 | 2.00E-04 |
| perinuclear region of cytoplasm | 46 | 6.3 | 2.20E-05 | 1.90E-03 |
| cytoplasmic vesicle | 23 | 3.2 | 8.50E-05 | 6.20E-03 |
| protein-DNA complex | 7 | 1 | 2.70E-04 | 1.70E-02 |
| cytoplasmic mRNA processing body | 11 | 1.5 | 6.80E-04 | 3.80E-02 |
| npBAF complex | 5 | 0.7 | 7.80E-04 | 3.90E-02 |
| early endosome | 20 | 2.7 | 1.10E-03 | 5.10E-02 |
| clathrin-coated vesicle | 9 | 1.2 | 1.40E-03 | 5.90E-02 |
| Golgi apparatus | 51 | 7 | 1.70E-03 | 6.50E-02 |
| late endosome | 13 | 1.8 | 2.20E-03 | 7.80E-02 |
| Golgi membrane | 37 | 5.1 | 3.30E-03 | 1.10E-01 |
| clathrin-coated pit | 8 | 1.1 | 4.40E-03 | 1.30E-01 |
| cell-cell adherens junction | 23 | 3.2 | 5.70E-03 | 1.60E-01 |
| cytoplasmic, membrane-bounded vesicle | 13 | 1.8 | 6.00E-03 | 1.60E-01 |
| transcription elongation factor complex | 5 | 0.7 | 1.00E-02 | 2.40E-01 |
| histone acetyltransferase complex | 5 | 0.7 | 1.00E-02 | 2.40E-01 |
| postsynaptic density | 15 | 2.1 | 1.00E-02 | 2.30E-01 |
| nuclear inclusion body | 4 | 0.5 | 1.10E-02 | 2.50E-01 |
| AP-2 adaptor complex | 4 | 0.5 | 1.10E-02 | 2.50E-01 |
| growth cone | 11 | 1.5 | 1.20E-02 | 2.50E-01 |
| myelin sheath | 13 | 1.8 | 1.30E-02 | 2.50E-01 |
| intracellular | 67 | 9.2 | 1.30E-02 | 2.50E-01 |
| cytoplasmic ribonucleoprotein granule | 5 | 0.7 | 1.30E-02 | 2.40E-01 |
| focal adhesion | 25 | 3.4 | 1.40E-02 | 2.40E-01 |
| nBAF complex | 4 | 0.5 | 1.40E-02 | 2.40E-01 |
| nuclear chromatin | 15 | 2.1 | 1.50E-02 | 2.40E-01 |
| nucleolus | 46 | 6.3 | 1.60E-02 | 2.50E-01 |
| integral component of endoplasmic reticulum membrane | 10 | 1.4 | 1.70E-02 | 2.50E-01 |
| SWI/SNF complex | 4 | 0.5 | 1.70E-02 | 2.50E-01 |
| lamellipodium | 13 | 1.8 | 1.80E-02 | 2.60E-01 |
| actin cytoskeleton | 16 | 2.2 | 1.80E-02 | 2.50E-01 |
| messenger ribonucleoprotein complex | 3 | 0.4 | 1.90E-02 | 2.50E-01 |
| synapse | 14 | 1.9 | 2.00E-02 | 2.60E-01 |
| ruffle | 9 | 1.2 | 2.00E-02 | 2.50E-01 |
| cytoplasmic vesicle membrane | 11 | 1.5 | 2.10E-02 | 2.50E-01 |
| vesicle | 11 | 1.5 | 2.30E-02 | 2.70E-01 |
| cell junction | 27 | 3.7 | 2.60E-02 | 2.90E-01 |
| nuclear membrane | 16 | 2.2 | 2.70E-02 | 3.00E-01 |
| terminal bouton | 7 | 1 | 2.90E-02 | 3.10E-01 |
| neuronal cell body | 20 | 2.7 | 3.00E-02 | 3.10E-01 |
| lysosomal membrane | 18 | 2.5 | 3.10E-02 | 3.10E-01 |
| autophagosome | 7 | 1 | 3.30E-02 | 3.30E-01 |
| intracellular ribonucleoprotein complex | 11 | 1.5 | 3.30E-02 | 3.20E-01 |
| SMAD protein complex | 3 | 0.4 | 3.40E-02 | 3.20E-01 |
| neurofilament | 3 | 0.4 | 3.40E-02 | 3.20E-01 |
| melanosome | 9 | 1.2 | 3.70E-02 | 3.40E-01 |
| axon | 15 | 2.1 | 4.20E-02 | 3.70E-01 |
| extrinsic component of cytoplasmic side of plasma membrane | 7 | 1 | 4.30E-02 | 3.70E-01 |
| cytoplasmic stress granule | 5 | 0.7 | 4.50E-02 | 3.80E-01 |
| **RT (Molecular Function)** | **Count** | **%** | ***P*-Value** | **FDR** |
| protein binding | 436 | 59.8 | 7.80E-13 | 6.90E-10 |
| protein kinase binding | 35 | 4.8 | 6.10E-06 | 2.70E-03 |
| thiol-dependent ubiquitin-specific protease activity | 14 | 1.9 | 1.40E-05 | 3.90E-03 |
| transcription factor activity, sequence-specific DNA binding | 66 | 9.1 | 1.40E-05 | 3.00E-03 |
| poly(A) RNA binding | 74 | 10.2 | 1.90E-05 | 3.20E-03 |
| transcription factor binding | 26 | 3.6 | 1.50E-04 | 2.20E-02 |
| DNA binding | 96 | 13.2 | 1.60E-04 | 2.00E-02 |
| sequence-specific DNA binding | 38 | 5.2 | 3.60E-04 | 3.90E-02 |
| RNA polymerase II core promoter proximal region sequence-specific DNA binding | 29 | 4 | 4.00E-04 | 3.80E-02 |
| transcriptional activator activity, RNA polymerase II core promoter proximal region sequence-specific binding | 21 | 2.9 | 1.10E-03 | 9.20E-02 |
| kinase activity | 21 | 2.9 | 1.40E-03 | 1.10E-01 |
| potassium ion binding | 5 | 0.7 | 1.70E-03 | 1.20E-01 |
| chromatin DNA binding | 9 | 1.2 | 1.90E-03 | 1.20E-01 |
| actin filament binding | 14 | 1.9 | 2.10E-03 | 1.30E-01 |
| protein kinase activity | 27 | 3.7 | 2.20E-03 | 1.20E-01 |
| RNA polymerase binding | 5 | 0.7 | 2.30E-03 | 1.20E-01 |
| ubiquitin protein ligase binding | 23 | 3.2 | 2.30E-03 | 1.10E-01 |
| cadherin binding involved in cell-cell adhesion | 23 | 3.2 | 2.60E-03 | 1.20E-01 |
| chromatin binding | 28 | 3.8 | 3.50E-03 | 1.50E-01 |
| RNA polymerase II distal enhancer sequence-specific DNA binding | 9 | 1.2 | 3.90E-03 | 1.60E-01 |
| Tat protein binding | 4 | 0.5 | 4.30E-03 | 1.60E-01 |
| histone acetyltransferase binding | 6 | 0.8 | 4.30E-03 | 1.60E-01 |
| Ras guanyl-nucleotide exchange factor activity | 12 | 1.6 | 5.70E-03 | 2.00E-01 |
| histone acetyltransferase activity (H4-K16 specific) | 4 | 0.5 | 5.90E-03 | 2.00E-01 |
| histone acetyltransferase activity (H4-K5 specific) | 4 | 0.5 | 5.90E-03 | 2.00E-01 |
| histone acetyltransferase activity (H4-K8 specific) | 4 | 0.5 | 5.90E-03 | 2.00E-01 |
| protein tyrosine kinase activity | 13 | 1.8 | 6.30E-03 | 2.00E-01 |
| ATP binding | 79 | 10.8 | 6.30E-03 | 1.90E-01 |
| core promoter sequence-specific DNA binding | 7 | 1 | 6.40E-03 | 1.90E-01 |
| histone deacetylase binding | 11 | 1.5 | 6.90E-03 | 1.90E-01 |
| protein serine/threonine kinase activity | 26 | 3.6 | 7.70E-03 | 2.10E-01 |
| mRNA 3'-UTR AU-rich region binding | 4 | 0.5 | 7.90E-03 | 2.10E-01 |
| ionotropic glutamate receptor binding | 5 | 0.7 | 8.40E-03 | 2.10E-01 |
| identical protein binding | 44 | 6 | 8.70E-03 | 2.10E-01 |
| protein homodimerization activity | 43 | 5.9 | 9.20E-03 | 2.20E-01 |
| transcription coactivator activity | 19 | 2.6 | 9.50E-03 | 2.20E-01 |
| double-stranded RNA binding | 8 | 1.1 | 9.80E-03 | 2.20E-01 |
| GTP binding | 26 | 3.6 | 9.90E-03 | 2.10E-01 |
| MAP kinase kinase kinase activity | 5 | 0.7 | 9.90E-03 | 2.10E-01 |
| voltage-gated chloride channel activity | 4 | 0.5 | 1.00E-02 | 2.10E-01 |
| sodium ion binding | 4 | 0.5 | 1.00E-02 | 2.10E-01 |
| transcription regulatory region DNA binding | 17 | 2.3 | 1.00E-02 | 2.10E-01 |
| GTPase activity | 18 | 2.5 | 1.10E-02 | 2.20E-01 |
| ion channel binding | 11 | 1.5 | 1.40E-02 | 2.60E-01 |
| RNA polymerase II transcription coactivator activity | 6 | 0.8 | 1.40E-02 | 2.60E-01 |
| transcriptional activator activity, RNA polymerase II distal enhancer sequence-specific binding | 5 | 0.7 | 1.60E-02 | 2.70E-01 |
| chloride ion binding | 4 | 0.5 | 1.60E-02 | 2.70E-01 |
| calmodulin binding | 15 | 2.1 | 1.80E-02 | 2.90E-01 |
| phosphatidylinositol binding | 9 | 1.2 | 1.90E-02 | 3.00E-01 |
| insulin-like growth factor receptor binding | 4 | 0.5 | 1.90E-02 | 3.10E-01 |
| phospholipid binding | 9 | 1.2 | 2.00E-02 | 3.10E-01 |
| thiol-dependent ubiquitinyl hydrolase activity | 8 | 1.1 | 2.00E-02 | 3.00E-01 |
| neurotrophin TRKA receptor binding | 3 | 0.4 | 2.10E-02 | 3.10E-01 |
| single-stranded RNA binding | 6 | 0.8 | 2.60E-02 | 3.70E-01 |
| protein heterodimerization activity | 28 | 3.8 | 2.90E-02 | 3.90E-01 |
| zinc ion binding | 60 | 8.2 | 3.00E-02 | 3.90E-01 |
| drug binding | 8 | 1.1 | 3.00E-02 | 3.90E-01 |
| insulin receptor binding | 5 | 0.7 | 3.20E-02 | 4.10E-01 |
| epidermal growth factor receptor binding | 5 | 0.7 | 3.20E-02 | 4.10E-01 |
| ubiquitin binding | 8 | 1.1 | 3.40E-02 | 4.20E-01 |
| protein self-association | 6 | 0.8 | 3.40E-02 | 4.10E-01 |
| DNA-dependent ATPase activity | 5 | 0.7 | 3.60E-02 | 4.20E-01 |
| AP-2 adaptor complex binding | 3 | 0.4 | 3.70E-02 | 4.30E-01 |
| RNA polymerase I CORE element sequence-specific DNA binding | 3 | 0.4 | 3.70E-02 | 4.30E-01 |
| core promoter binding | 7 | 1 | 4.00E-02 | 4.50E-01 |
| 1-phosphatidylinositol binding | 4 | 0.5 | 4.20E-02 | 4.60E-01 |
| mRNA 3'-UTR binding | 6 | 0.8 | 4.30E-02 | 4.60E-01 |
| Lys48-specific deubiquitinase activity | 3 | 0.4 | 4.60E-02 | 4.80E-01 |
| metal ion binding | 97 | 13.3 | 4.60E-02 | 4.80E-01 |
| repressing transcription factor binding | 5 | 0.7 | 4.80E-02 | 4.80E-01 |
| translation initiation factor binding | 4 | 0.5 | 4.80E-02 | 4.80E-01 |

**Supplementary Table 4** KEGG pathways analysis of the predicted target genes affected by the six prognostic miRNAs

| **Pathway** | **Count** | **%** | ***P*-Value** | **FDR** |
| --- | --- | --- | --- | --- |
| FoxO signaling pathway | 19 | 2.6 | 5.70E-06 | 1.30E-03 |
| Pancreatic cancer | 12 | 1.6 | 4.60E-05 | 5.20E-03 |
| Neurotrophin signaling pathway | 16 | 2.2 | 8.10E-05 | 6.20E-03 |
| Transcriptional misregulation in cancer | 19 | 2.6 | 1.20E-04 | 7.10E-03 |
| Axon guidance | 16 | 2.2 | 1.50E-04 | 7.10E-03 |
| Pathways in cancer | 32 | 4.4 | 2.00E-04 | 7.70E-03 |
| MAPK signaling pathway | 24 | 3.3 | 2.10E-04 | 6.90E-03 |
| PI3K-Akt signaling pathway | 29 | 4 | 2.60E-04 | 7.40E-03 |
| Ras signaling pathway | 21 | 2.9 | 6.90E-04 | 1.80E-02 |
| Colorectal cancer | 10 | 1.4 | 7.40E-04 | 1.70E-02 |
| Proteoglycans in cancer | 19 | 2.6 | 1.00E-03 | 2.20E-02 |
| Endocytosis | 22 | 3 | 1.50E-03 | 2.80E-02 |
| Endocrine and other factor-regulated calcium reabsorption | 8 | 1.1 | 1.90E-03 | 3.30E-02 |
| ErbB signaling pathway | 11 | 1.5 | 2.40E-03 | 3.80E-02 |
| Prostate cancer | 11 | 1.5 | 2.60E-03 | 3.90E-02 |
| Oocyte meiosis | 12 | 1.6 | 4.10E-03 | 5.40E-02 |
| Renal cell carcinoma | 9 | 1.2 | 4.20E-03 | 5.20E-02 |
| Aldosterone-regulated sodium reabsorption | 7 | 1 | 4.20E-03 | 5.00E-02 |
| Epithelial cell signaling in Helicobacter pylori infection | 9 | 1.2 | 5.00E-03 | 5.60E-02 |
| Hepatitis B | 14 | 1.9 | 5.30E-03 | 5.60E-02 |
| Fc epsilon RI signaling pathway | 9 | 1.2 | 5.50E-03 | 5.60E-02 |
| HIF-1 signaling pathway | 11 | 1.5 | 5.70E-03 | 5.50E-02 |
| Non-small cell lung cancer | 8 | 1.1 | 6.60E-03 | 6.20E-02 |
| Small cell lung cancer | 10 | 1.4 | 6.70E-03 | 6.00E-02 |
| HTLV-I infection | 20 | 2.7 | 6.80E-03 | 5.90E-02 |
| Choline metabolism in cancer | 11 | 1.5 | 7.00E-03 | 5.80E-02 |
| Adherens junction | 9 | 1.2 | 7.20E-03 | 5.70E-02 |
| T cell receptor signaling pathway | 11 | 1.5 | 8.00E-03 | 6.20E-02 |
| mTOR signaling pathway | 8 | 1.1 | 8.00E-03 | 6.00E-02 |
| Insulin signaling pathway | 13 | 1.8 | 9.20E-03 | 6.60E-02 |
| GnRH signaling pathway | 10 | 1.4 | 1.00E-02 | 7.20E-02 |
| Cell cycle | 12 | 1.6 | 1.10E-02 | 7.20E-02 |
| Sphingolipid signaling pathway | 11 | 1.5 | 2.20E-02 | 1.40E-01 |
| Progesterone-mediated oocyte maturation | 9 | 1.2 | 2.30E-02 | 1.40E-01 |
| Bladder cancer | 6 | 0.8 | 2.30E-02 | 1.40E-01 |
| Signaling pathways regulating pluripotency of stem cells | 12 | 1.6 | 2.50E-02 | 1.40E-01 |
| Chronic myeloid leukemia | 8 | 1.1 | 2.50E-02 | 1.40E-01 |
| TNF signaling pathway | 10 | 1.4 | 2.60E-02 | 1.40E-01 |
| Insulin resistance | 10 | 1.4 | 2.90E-02 | 1.50E-01 |
| Vasopressin-regulated water reabsorption | 6 | 0.8 | 3.00E-02 | 1.60E-01 |
| Mineral absorption | 6 | 0.8 | 3.60E-02 | 1.80E-01 |
| Bacterial invasion of epithelial cells | 8 | 1.1 | 3.60E-02 | 1.80E-01 |
| Focal adhesion | 15 | 2.1 | 3.70E-02 | 1.80E-01 |
| Osteoclast differentiation | 11 | 1.5 | 3.70E-02 | 1.80E-01 |
| Synaptic vesicle cycle | 7 | 1 | 4.00E-02 | 1.80E-01 |
| Regulation of actin cytoskeleton | 15 | 2.1 | 4.40E-02 | 2.00E-01 |
| Glycosaminoglycan biosynthesis - chondroitin sulfate / dermatan sulfate | 4 | 0.5 | 4.40E-02 | 1.90E-01 |
| Glioma | 7 | 1 | 4.50E-02 | 1.90E-01 |

**Supplementary Table 5** Association between 6-miRNA risk score and clinicopathological features of spinal chordoma patients in the training cohort (n = 54)^a^

| Clinicopathological factors | No. of patients (n = 54) | miRNA risk score | | | Test statistic | *P-*value |
| --- | --- | --- | --- | --- | --- | --- |
|  |  | Low (%) | High (%) | |  |  |
| Age (years) |  |  | |  |  |  |
| ≤ 50 | 23 | 7 (30.4%) | | 16 (69.6%) | 0.396 | 0.529 |
| > 50 | 31 | 12 (38.7%) | | 19 (61.3%) |  |  |
| Sex |  |  | |  |  |  |
| Male | 35 | 11 (31.4%) | | 24 (68.6%) | 0.616 | 0.433 |
| Female | 19 | 8 (42.1%) | | 11 (57.9%) |  |  |
| Tumor size |  |  | |  |  |  |
| ≤ 5 cm | 21 | 6 (28.6%) | | 15 (71.4%) | 0.659 | 0.417 |
| > 5 cm | 33 | 13 (39.4%) | | 20 (60.6%) |  |  |
| Tumor location |  |  | |  |  |  |
| Sacral vertebra | 42 | 17 (40.5%) | | 25 (59.5%) | 1.394 | 0.238 |
| Mobile spine | 12 | 2 (16.7%) | | 10 (83.3%) |  |  |
| Surrounding  muscle invasion |  |  | |  |  |  |
| Yes | 36 | 9 (25%) | | 27 (75%) | 4.913 | **0.027** |
| No | 18 | 10 (55.6%) | | 8 (44.4%) |  |  |
| Preoperative recurrence |  |  | |  |  |  |
| Yes | 11 | 4 (36.4%) | | 7 (63.6%) | 0.000 | 1.000 |
| No | 43 | 15 (34.9%) | | 28 (65.1%) |  |  |
| Grade |  |  | |  |  |  |
| High | 38 | 13 (34.2%) | | 25 (65.8%) | 0.053 | 0.817 |
| Low | 16 | 6 (37.5%) | | 10 (62.5%) |  |  |
| Enneking staging |  |  | |  |  |  |
| IA + IB + IIA | 23 | 11 (47.8%) | | 12 (52.2%) | 2.807 | 0.094 |
| IIB + III | 31 | 8 (25.8%) | | 23 (74.2%) |  |  |
| Type of surgery |  |  | |  |  |  |
| EI | 18 | 4 (22.2%) | | 14 (77.8%) | 1.989 | 0.158 |
| EA | 36 | 15 (41.7%) | | 21 (58.3%) |  |  |
| Tumor hemorrhage |  |  | |  |  |  |
| No | 10 | 2 (20%) | | 8 (80%) | 0.558 | 0.455 |
| Yes | 44 | 17 (38.6%) | | 27 (61.4%) |  |  |
| Tumor necrosis |  |  | |  |  |  |
| Absent + Mild | 31 | 12 (38.7%) | | 19 (61.3%) | 0.396 | 0.528 |
| Moderate + Severe | 23 | 7 (30.4%) | | 16 (69.6%) |  |  |
| Ki-67 index |  |  | |  |  |  |
| Low | 25 | 11 (44%) | | 14 (56%) | 1.586 | 0.208 |
| High | 29 | 8 (27.6%) | | 21 (72.4%) |  |  |
| Tumor PD-L1 |  |  | |  |  |  |
| Positive | 37 | 10 (27%) | | 27 (73%) | 3.430 | 0.064 |
| Negative | 17 | 9 (52.9%) | | 8 (47.1%) |  |  |
| Lobular pattern growth |  |  | |  |  |  |
| Yes | 21 | 6 (28.6%) | | 15 (71.4%) | 0.659 | 0.417 |
| No | 33 | 13 (39.4%) | | 20 (60.6%) |  |  |
| Overall TILs |  |  | |  |  |  |
| Positive | 31 | 11 (35.5%) | | 20 (64.5%) | 0.003 | 0.957 |
| Negative | 23 | 8 (34.8%) | | 15 (65.2%) |  |  |

Bold indicates *P* < 0.05.

EI, Enneking inappropriate; EA, Enneking appropriate; TILs, tumor-infiltrating lymphocytes; PD-L1,

programmed cell death-1 ligand 1; ^a^analyzed by the Chi-square test.

**Supplementary Table 6** Association between 6-miRNA risk score and clinicopathological features of spinal chordoma patients in the validation cohort (n = 60)^a^

| Clinicopathological factors | No. of patients (n = 60) | miRNA risk score | | | Test statistic | *P-*value |
| --- | --- | --- | --- | --- | --- | --- |
|  |  | Low (%) | High (%) | |  |  |
| Age (years) |  |  | |  |  |  |
| ≤ 50 | 20 | 13 (65%) | | 7 (35%) | 2.136 | 0.144 |
| > 50 | 40 | 18 (45%) | | 22 (55%) |  |  |
| Sex |  |  | |  |  |  |
| Male | 42 | 21 (50%) | | 21 (50%) | 0.156 | 0.693 |
| Female | 18 | 10 (55.6%) | | 8 (44.4%) |  |  |
| Tumor size |  |  | |  |  |  |
| ≤ 5 cm | 22 | 11 (50%) | | 11 (50%) | 0.039 | 0.844 |
| > 5 cm | 38 | 20 (52.6%) | | 18 (47.4%) |  |  |
| Tumor location |  |  | |  |  |  |
| Sacral vertebra | 45 | 22 (48.9%) | | 23 (51.1%) | 0.556 | 0.456 |
| Mobile spine | 15 | 9 (60%) | | 6 (40%) |  |  |
| Surrounding  muscle invasion |  |  | |  |  |  |
| Yes | 31 | 23 (74.2%) | | 8 (25.8%) | 13.033 | **< 0.001** |
| No | 29 | 8 (27.6%) | | 21 (72.4%) |  |  |
| Preoperative recurrence |  |  | |  |  |  |
| Yes | 13 | 6 (46.2%) | | 7 (53.8%) | 0.202 | 0.653 |
| No | 47 | 25 (53.2%) | | 22 (46.8%) |  |  |
| Grade |  |  | |  |  |  |
| High | 42 | 17 (40.5%) | | 25 (59.5%) | 7.020 | **0.008** |
| Low | 18 | 14 (77.8%) | | 4 (22.2%) |  |  |
| Enneking staging |  |  | |  |  |  |
| IA + IB + IIA | 34 | 22 (64.7%) | | 12 (35.3%) | 5.342 | **0.021** |
| IIB + III | 26 | 9 (34.6%) | | 17 (65.4%) |  |  |
| Type of surgery |  |  | |  |  |  |
| EI | 24 | 10 (41.7%) | | 14 (58.3%) | 1.602 | 0.206 |
| EA | 36 | 21 (58.3%) | | 15 (41.7%) |  |  |
| Tumor hemorrhage |  |  | |  |  |  |
| No | 17 | 16 (94.1%) | | 1 (5.9%) | 17.118 | **< 0.001** |
| Yes | 43 | 15 (34.9%) | | 28 (65.1%) |  |  |
| Tumor necrosis |  |  | |  |  |  |
| Absent + Mild | 33 | 20 (60.6%) | | 13 (39.4%) | 2.347 | 0.126 |
| Moderate + Severe | 27 | 11 (40.7%) | | 16 (59.3%) |  |  |
| Ki-67 index |  |  | |  |  |  |
| Low | 26 | 20 (76.9%) | | 6 (23.1%) | 11.720 | **0.001** |
| High | 34 | 11 (32.4%) | | 23 (67.6%) |  |  |
| Tumor PD-L1 |  |  | |  |  |  |
| Positive | 40 | 20 (50%) | | 20 (50%) | 0.133 | 0.715 |
| Negative | 20 | 11 (55%) | | 9 (45%) |  |  |
| Lobular pattern growth |  |  | |  |  |  |
| Yes | 25 | 15 (60%) | | 10 (40%) | 1.192 | 0.275 |
| No | 35 | 16 (45.7%) | | 19 (54.3%) |  |  |
| Overall TILs |  |  | |  |  |  |
| Positive | 34 | 17 (50%) | | 17 (50%) | 0.087 | 0.768 |
| Negative | 26 | 14 (53.8%) | | 12 (46.2%) |  |  |

Bold indicates *P* < 0.05.

EI, Enneking inappropriate; EA, Enneking appropriate; TILs, tumor-infiltrating lymphocytes; PD-L1,

programmed cell death-1 ligand 1; ^a^analyzed by the Chi-square test.


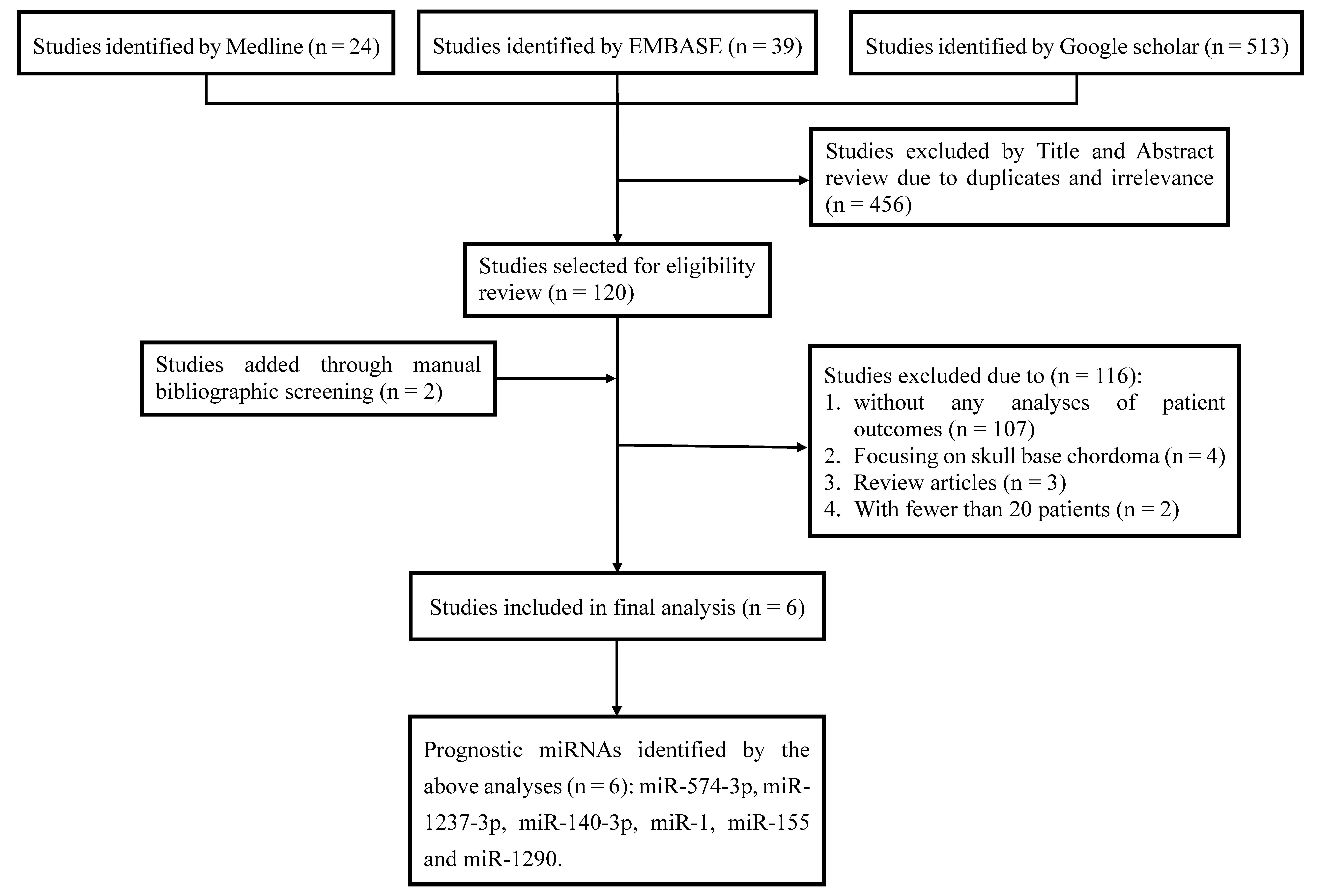


**Supplementary Fig. 1** Flow diagram of literature search showing studies identified, included and excluded at each stage.


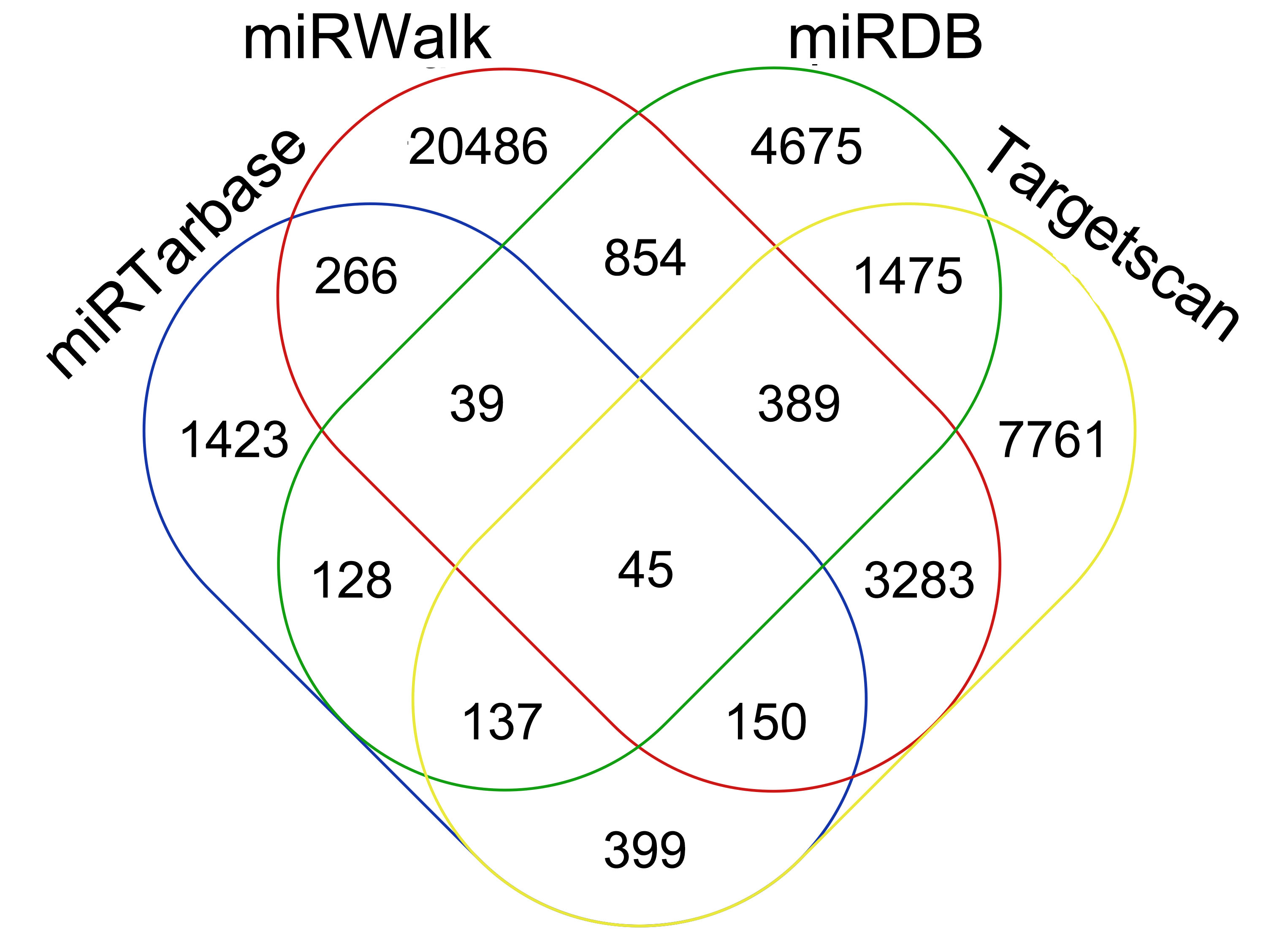


**Supplementary Fig. 2** Predicted target genes of the six prognostic microRNAs using four online complementary databases. Genes were selected for subsequent analysis only when they were jointly predicted by algorithms of at least three databases.


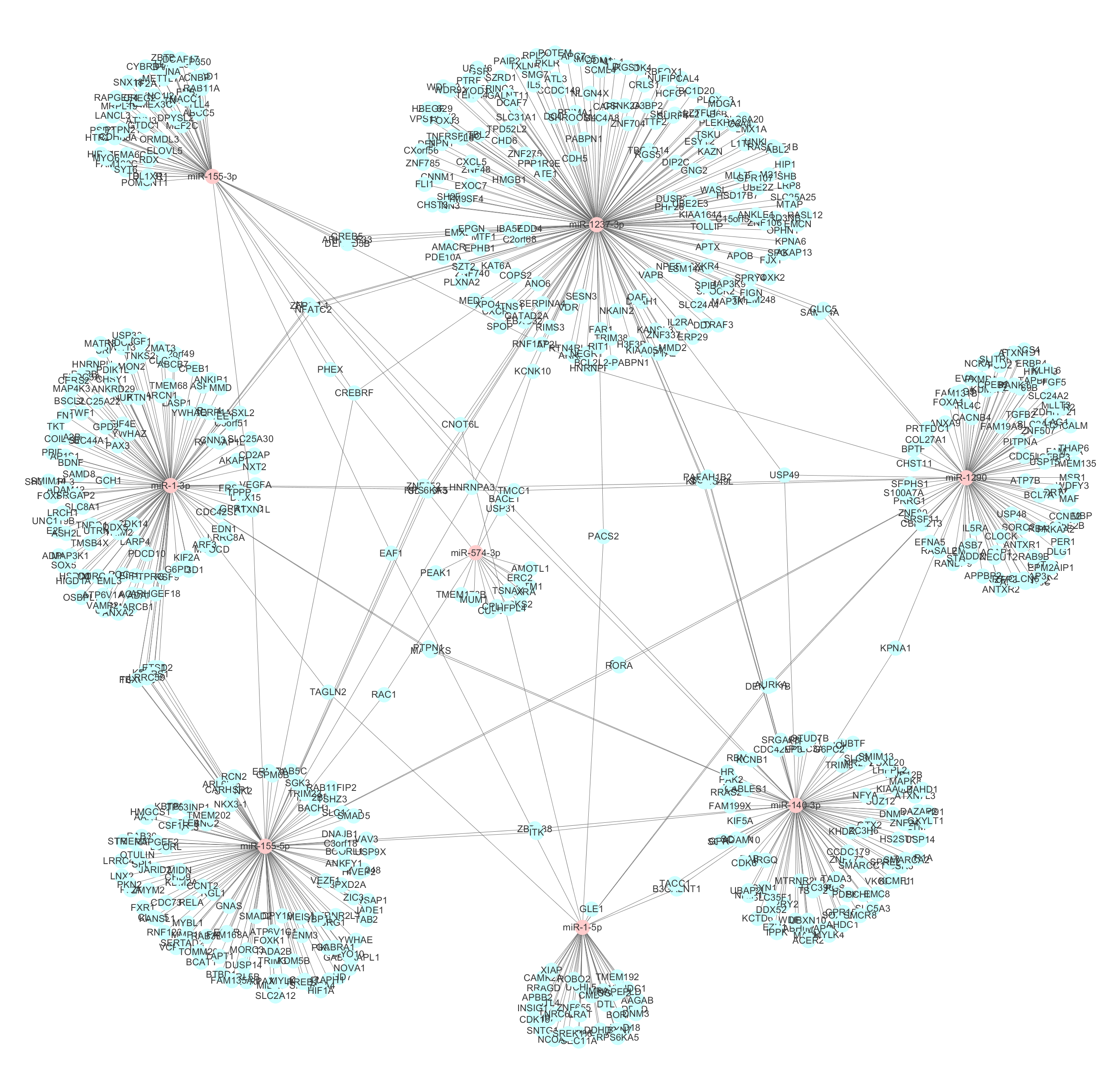


**Supplementary Fig. 3** Visualization of microRNA-target regulatory network using the Cytoscape version 3.5.1.


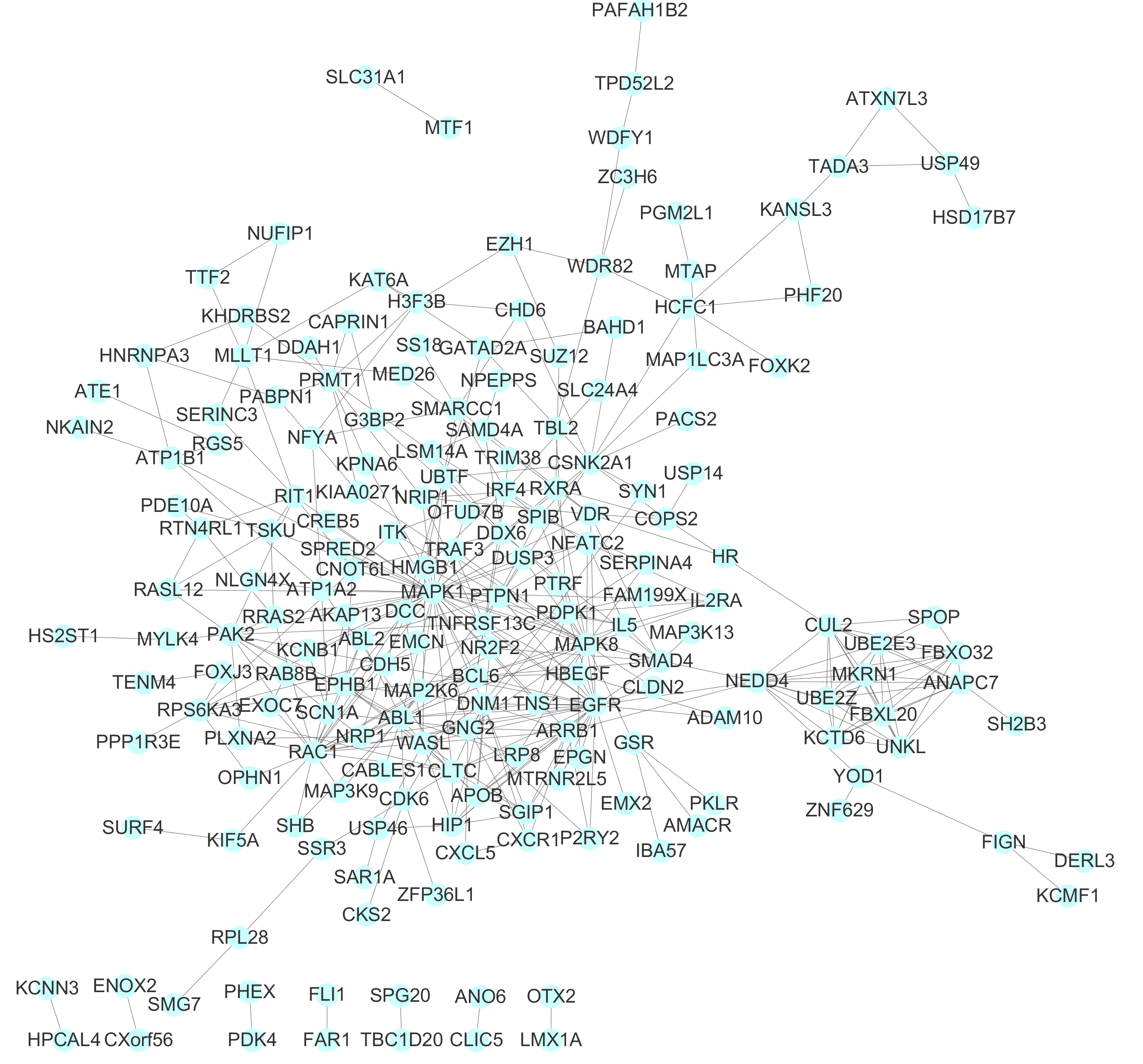


**Supplementary Fig. 4** Visualization of protein-protein interaction network using the STRING database.





**Supplementary Fig. 5** Cutoff determination for the six prognostic microRNAs in prognosis analysis with the overall survival as the outcome parameter.


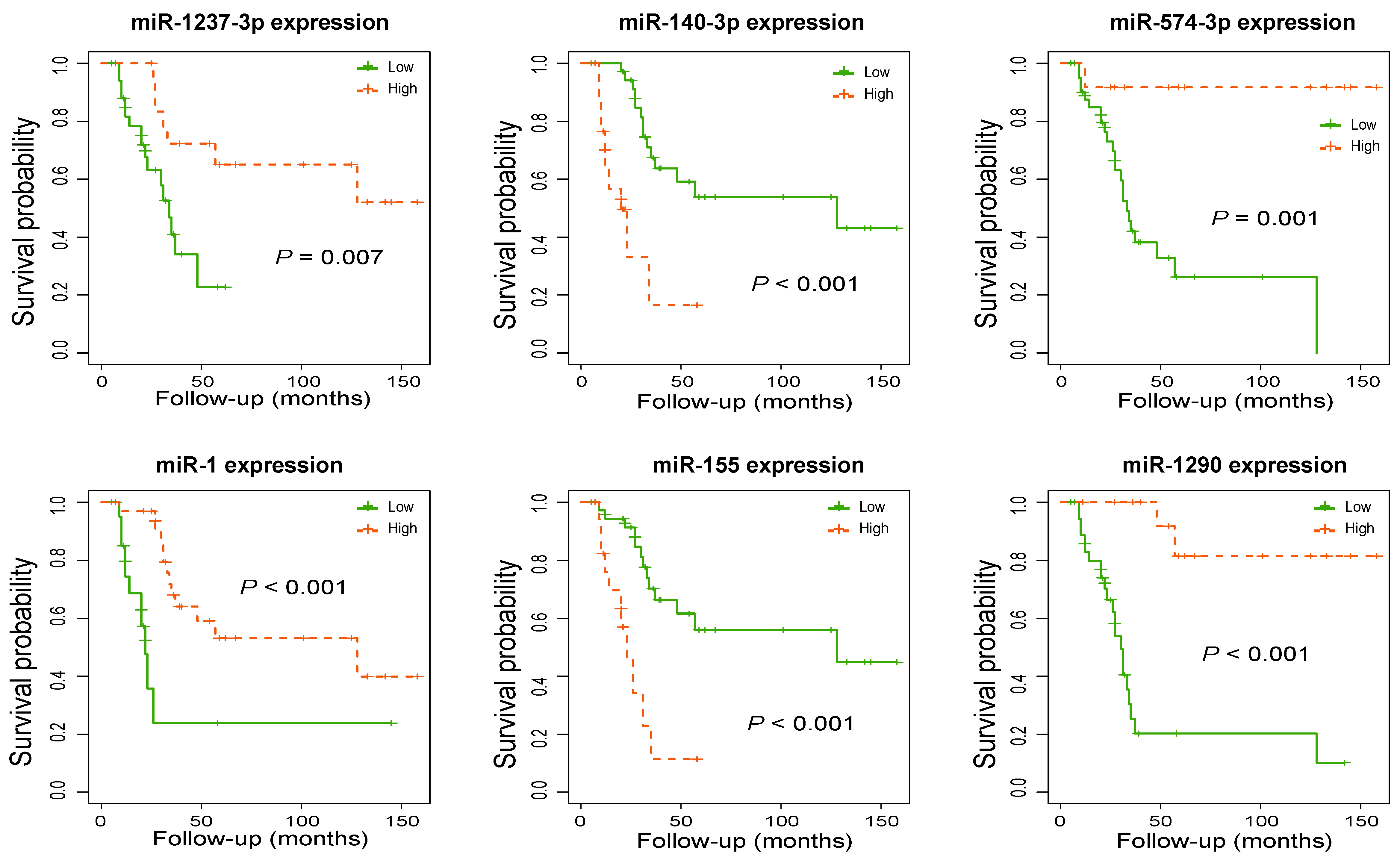


**Supplementary Fig. 6** Kaplan-Meier survival analysis of OS for 54 spinal chordoma patients in the training cohort according to the six prognostic microRNAs selected for subsequent miRscore construction. OS, overall survival.


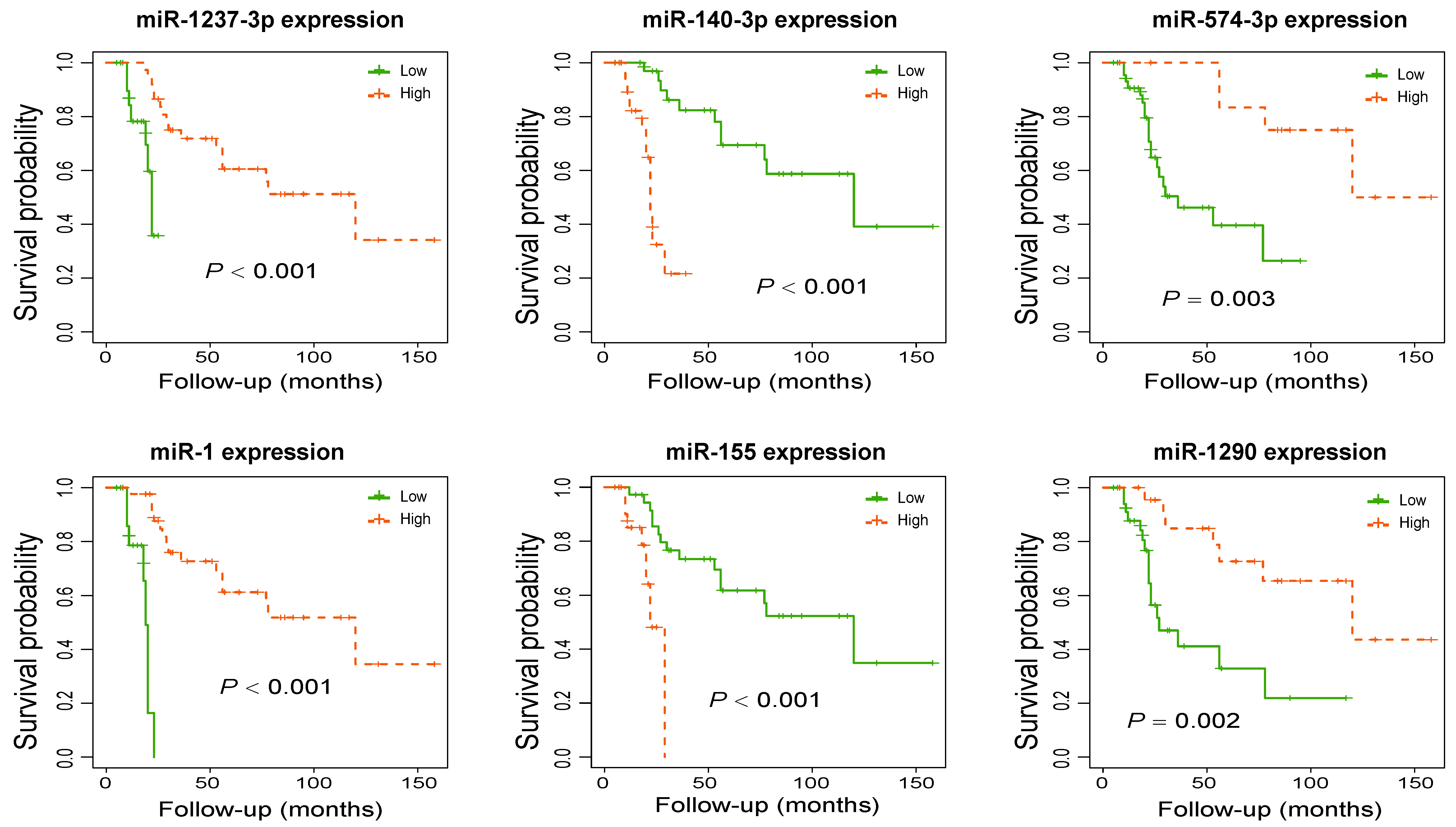


**Supplementary Fig. 7** Kaplan-Meier survival analysis of OS for 60 spinal chordoma patients in the validation cohort according to the six prognostic microRNAs selected for subsequent miRscore construction. OS, overall survival.


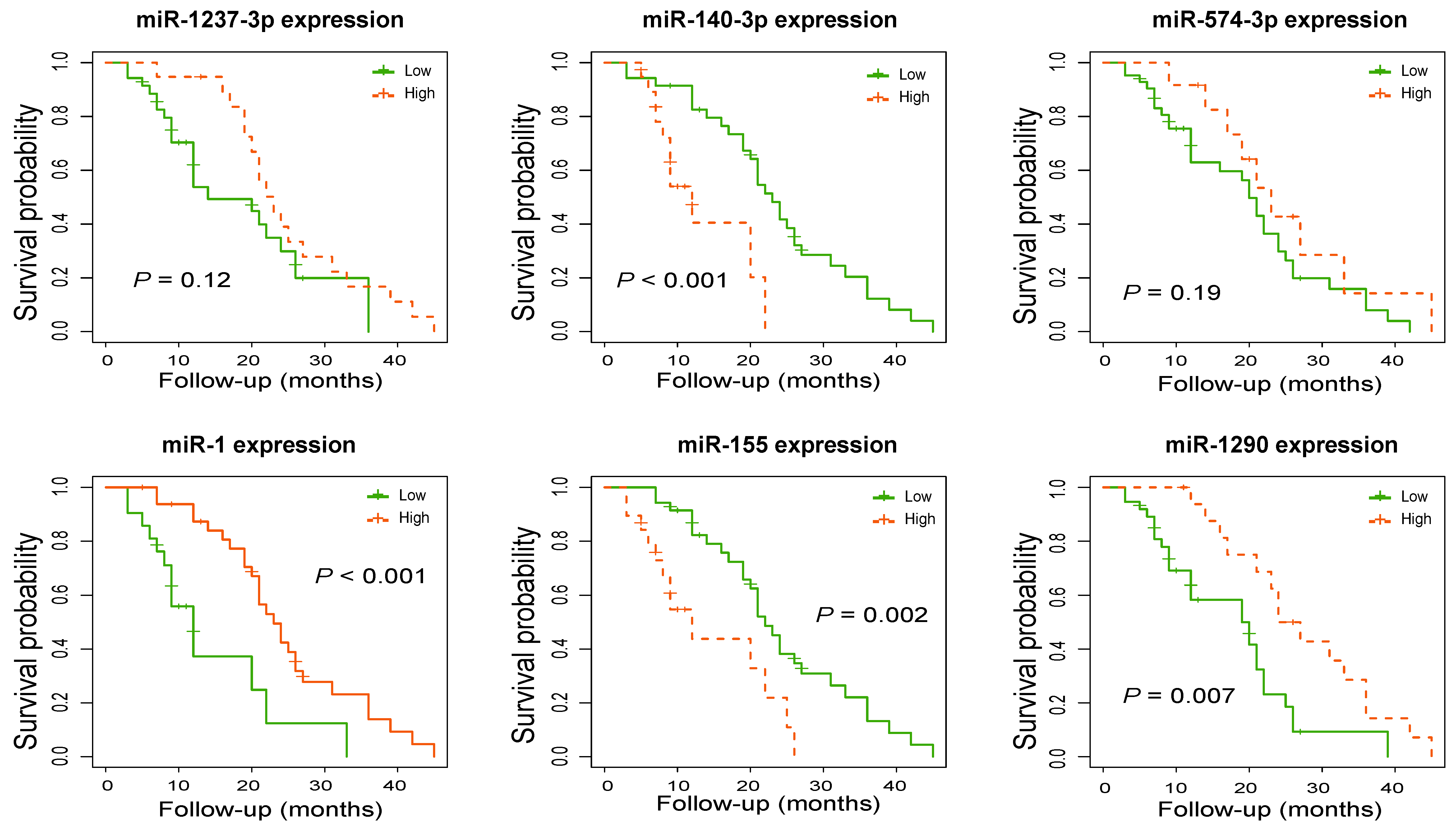


**Supplementary Fig. 8** Kaplan-Meier survival analysis of LRFS for 54 spinal chordoma patients in the training cohort according to the six prognostic microRNAs selected for subsequent miRscore construction. LRFS, local recurrence-free survival.


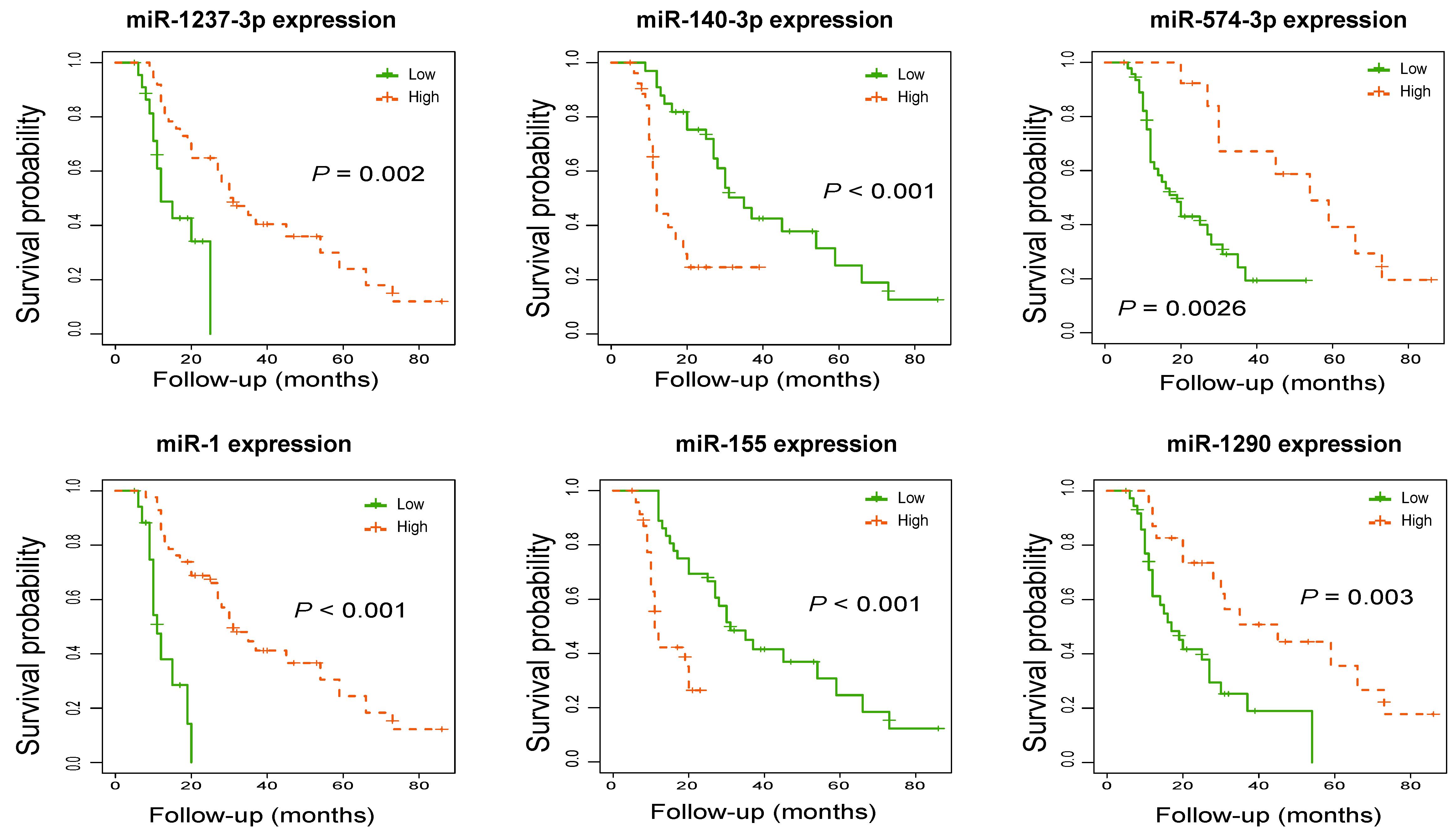


**Supplementary Fig. 9** Kaplan-Meier survival analysis of LRFS for 60 spinal chordoma patients in the validation cohort according to the six prognostic microRNAs selected for subsequent miRscore construction. LRFS, local recurrence-free survival.
